# Revealing uncertainty in the status of biodiversity change

**DOI:** 10.1038/s41586-024-07236-z

**Published:** 2024-03-27

**Authors:** T. F. Johnson, A. P. Beckerman, D. Z. Childs, T. J. Webb, K. L. Evans, C. A. Griffiths, P. Capdevila, C. F. Clements, M. Besson, R. D. Gregory, G. H. Thomas, E. Delmas, R. P. Freckleton

**Affiliations:** 1 School of Biosciences, Ecology and Evolutionary Biology, University of Sheffield, Sheffield, UK; 2 School of Biological Sciences, Biosciences, University of Bristol, Bristol, UK; 3 Departament de Biologia Evolutiva, Ecologia i Ciències Ambientals, Universitat de Barcelona (UB), Barcelona, Spain; 4 Institut de Recerca de la Biodiversitat (IRBio), Universitat de Barcelona (UB), Barcelona, Spain; 5 RSPB Centre for Conservation Science, The Lodge, Sandy, UK; 6 Centre for Biodiversity & Environment Research, Department of Genetics, Evolution and Environment, University College London, London, UK; 7 Habitat, Montreal, Quebec Canada; 8 Institut des Sciences de la Forêt Tempérée, Université du Québec en Outaouais, Ripon, Quebec Canada; 9 Debrecen Biodiversity Centre, University of Debrecen, Debrecen, Hungary; 10 Present Address: Swedish University of Agricultural Sciences, Department of Aquatic Resources, Institute of Marine Research, Lysekil, Sweden; 11 Present Address: Sorbonne Université, CNRS, Biologie Intégrative des Organismes Marins, BIOM, Banyuls-sur-Mer, France

**Keywords:** Biodiversity, Ecological modelling, Biogeography, Phylogenetics, Evolutionary ecology

## Abstract

Biodiversity faces unprecedented threats from rapid global change^1^. Signals of biodiversity change come from time-series abundance datasets for thousands of species over large geographic and temporal scales. Analyses of these biodiversity datasets have pointed to varied trends in abundance, including increases and decreases. However, these analyses have not fully accounted for spatial, temporal and phylogenetic structures in the data. Here, using a new statistical framework, we show across ten high-profile biodiversity datasets^2–11^ that increases and decreases under existing approaches vanish once spatial, temporal and phylogenetic structures are accounted for. This is a consequence of existing approaches severely underestimating trend uncertainty and sometimes misestimating the trend direction. Under our revised average abundance trends that appropriately recognize uncertainty, we failed to observe a single increasing or decreasing trend at 95% credible intervals in our ten datasets. This emphasizes how little is known about biodiversity change across vast spatial and taxonomic scales. Despite this uncertainty at vast scales, we reveal improved local-scale prediction accuracy by accounting for spatial, temporal and phylogenetic structures. Improved prediction offers hope of estimating biodiversity change at policy-relevant scales, guiding adaptive conservation responses.

## Abstract (web summary)

This study presents an approach to deal with spatial, temporal and phylogenetic non-independence in large-scale analyses of biodiversity change, improving trend estimation and inference across scales.

## Main

Accelerating rates of species extinction are driving global changes in biodiversity, threatening ecosystems and the services they provide^1^. In an attempt to reverse biodiversity declines, world leaders, policymakers and academics have called for action^12^. Evidence-based actions require long-term datasets and rigorous modelling to reliably detect and attribute biodiversity change through time^13,14^. At present, some of the most influential estimates of biodiversity change are calculated using datasets such as BioTIME^2^, the Living Planet^15^ or the North American Breeding Bird Survey^3^. Inferences from these abundance datasets have shaped policy^16^ and are considered by some to be a key pillar of global biodiversity monitoring^17^.

Biodiversity datasets are complex and typically subject to one or more sources of non-independence across the axes of time, space and evolution. This presents a challenge for analysis, as omission of even one of these sources of non-independence from a statistical model can lead to underestimation of uncertainty, incorrect trends and poorly resolved prediction, and ultimately undermines current interpretation of wildlife abundance trends^18–20^. A unifying feature of previous studies is that they are characterized by the consistent omission of one or more of these dependencies from their analysis. This imposes a risk that past estimates of abundance change—pointing to declines^15,21^, no net change^18,22,23^ and recovery^24^—may be unreliable.

**Fig. 1:**
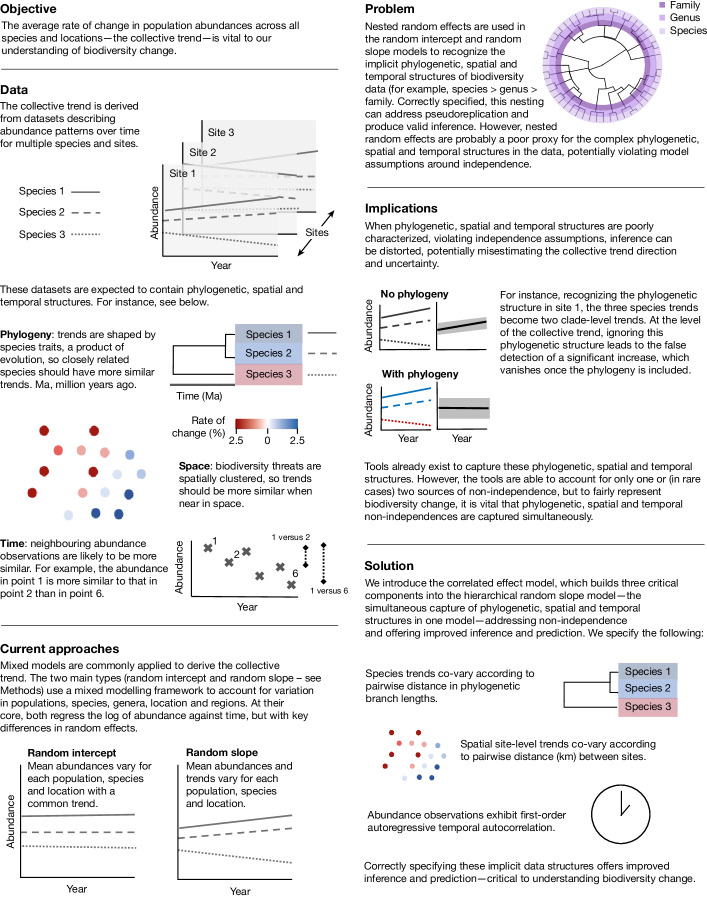
Impact of correlative non-independence on collective abundance trends. The text and images show the objective, implicit and key features of large-scale abundance datasets, current approaches for analysis, the problem, its implications and the solution.

Non-independence can be classified in a variety of ways, which we split into two core types: hierarchical, for which observations are pseudoreplicated or nested (for example, multiple trends for a given species, site or region in time); and correlative, for which observations become increasingly correlated (sometimes termed autocorrelation) when close in time^25^, space^26^ or phylogeny^27^. Under correlative non-independence, we may expect sequential abundance values in a time series to be more similar, and trends should be similar when near in space or in closely related species (Fig. 1). Although studies commonly account for hierarchical non-independence using features such as random effects in mixed models, a literature review covering hundreds of papers published in high-impact journals since 2010 revealed that studies rarely account for correlative non-independence across space (accounted for in 7% of studies), phylogeny (14%) or time (32%; Supplementary Table 1). Further, no biodiversity model has yet been formalized to account for all three sources of correlative non-independence at the same time.

Here we show that ignoring non-independence has serious consequences for inference of biodiversity trends. We introduce the correlated effect model, which incorporates hierarchical non-independence and all three sources of correlative non-independence, and apply it to ten high-profile, multi-species datasets that have been used to infer abundance trends in global biodiversity^2–11^. Combined, these datasets describe the abundance (including relative abundance and densities) patterns of more than 30,000 populations, representing about 3,100 species and about 6,000 unique locations, and are considered some of the best biodiversity monitoring datasets available.

## Non-independence increases uncertainty

**Fig. 2:**
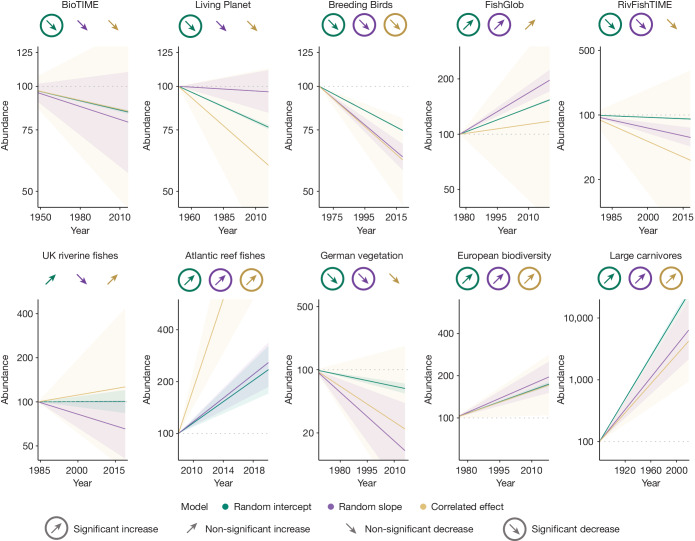
Widely used statistical models misrepresent biodiversity abundance trends. Abundance trend projections across ten high-profile datasets under three different models. Circles represent the collective trend (the coefficient describing the change in abundance over time averaged across all species and locations) for each dataset in our three models (from left to right): random intercept, random slope and the correlated effect model that simultaneously accounts for temporal, spatial and phylogenetic correlative non-independence. We specify four categories of trend: significant increase—coefficient is positive and significant; non-significant increase—coefficient is positive but not significant (that is, no detectable change); non-significant decrease—coefficient is negative but not significant (that is, no detectable change); significant decrease—coefficient is negative and significant. Significance indicates that the coefficient does not overlap zero at a 50% credible interval. Coefficients and 95% credible intervals are available in Supplementary Table 4. We use the collective trend coefficient and 50% credible intervals (represented by shading) to produce abundance projections for each model in each dataset from an arbitrary baseline abundance of 100. Abundance projections cover the time span of the observed data and are presented on the log_10_ scale. Source Data

We compared our correlated effect model with two mixed-effect modelling frameworks that are commonly used and account only for hierarchical non-independence: random intercept and random slope (both described in Fig. 1). Across the 44 relevant studies identified in a literature search spanning 282 published papers, 43% (*n* = 19) used a version of the random intercept model and 50% (*n* = 22) used a version of the random slope model (Supplementary Table 1). Comparing these commonly applied approaches to the correlated effect model, we detect a pronounced shift in collective abundance trends (that is, the model-derived average rate of change in abundance across all species and locations), and show that existing approaches underestimate collective trend uncertainty and can misestimate direction (Fig. 2).

Collective abundance trend uncertainty (that is, the standard deviation (s.d.) around the abundance–time coefficient) was underestimated in all ten datasets in both the random intercept and random slope models. These underestimates are large, with uncertainty in the correlated effect model 26 times greater [95% confidence interval (CI): 14–47] than that in the random intercept model and 3.4 times greater [95% CI: 1.8–6.2] than that in the random slope model. Further, after accounting for correlative non-independence, we find instances in which the trend direction shifts and even reverses (for example, from negative to positive). For instance, in the Living Planet dataset, a decreasing trend in the random intercept model shifts to a stable trend in the random slope model, before shifting back to a sharp albeit uncertain decrease after accounting for correlative non-independence. In three databases—the Living Planet, RivFishTIME and Atlantic reef fishes—the mean trends were more extreme under the correlated effect model, shifting away from zero (that is, no net change in abundance), although still highly uncertain. Across the three models, we observed complete agreement in trend direction and significance status (50% credible intervals) in only four of the datasets. At 95% credible intervals, we found no instances in which models agreed on trend direction and significance status.

**Extended Data Fig. 1:**
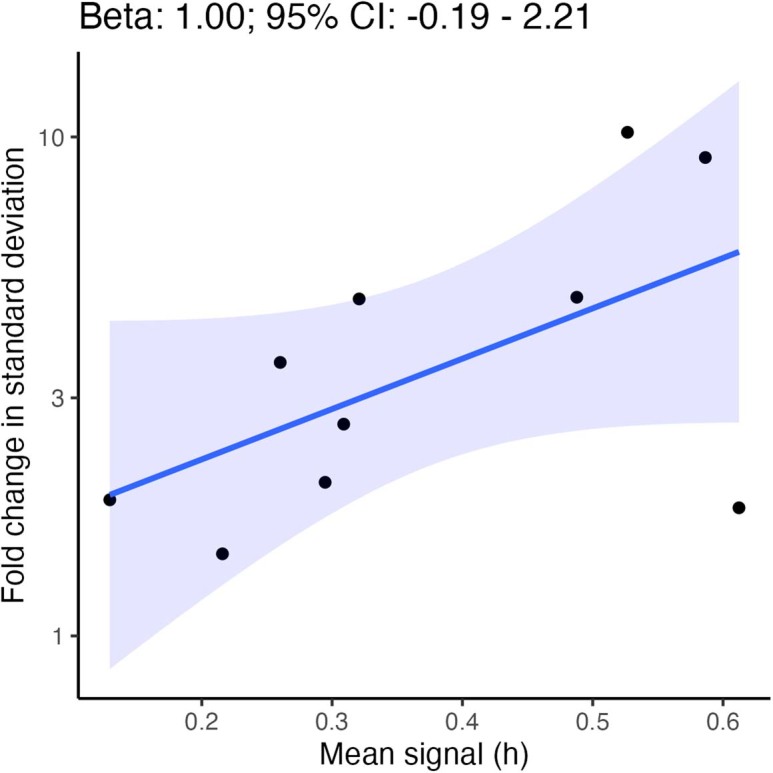
Impact of phylogenetic and spatial signal on inference. Fold change in the standard deviation of the abundance-time coefficient between the correlated effect and random slopes model, plotted against the mean signal. Mean signal is calculated by finding the mean of the phylogenetic signal (variance captured by the phylogeny divided by the sum of the phylogenetic and non-phylogenetic variance) and spatial signal (as in the phylogeny). n = 10 with each point representing a dataset. Shading represents 95% confidence intervals from a linear model.

**Extended Data Fig. 2:**
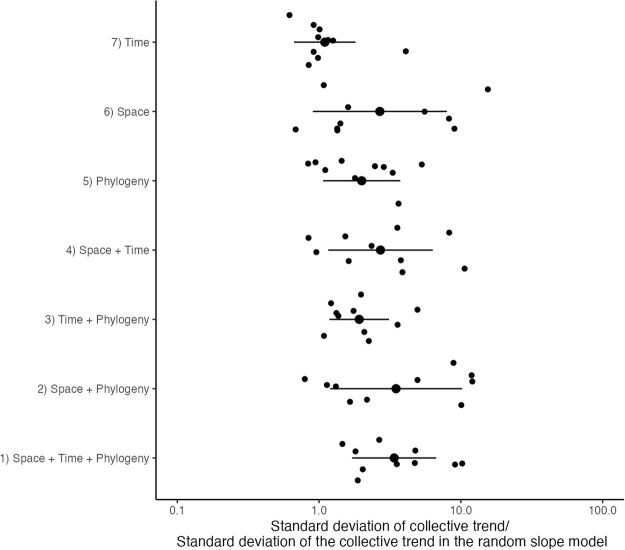
Contribution of spatial, temporal and phylogenetic components to collective trend uncertainty. Relative fold change in the standard deviation of the collective trend as additional components (space, time or phylogeny) are added to the random slope model. Models are compared to the standard deviation of the collective trend in the random slope model. In each model comparison (y-axis), n = 10 with each point representing a dataset. The larger point and error bar represents the mean change and associated standard deviation around this mean.

Collective abundance trend uncertainty is likely to be underestimated when hierarchical terms (for example, random effects) fail to effectively represent the complex spatial, phylogenetic and temporal structures in the data (Extended Data Fig. 1). This is an apparently common phenomenon given all ten datasets underestimate uncertainty, and across the ten datasets, we find that correlative terms proportionally account for approximately one-third of the variation in the data (spatial: mean = 0.34 s.d. = 0.3; phylogeny: mean = 0.41, s.d. = 0.28), relative to the combined variance captured by the respective hierarchical and correlated terms. There is no comparable metric for the temporal term that describes the correlation between abundances instead of covariance between trends. Notably, the stark increase in uncertainty is not a consequence of simply introducing additional correlated terms. This is because uncertainty tends to increase substantially only when the correlated terms are capturing a high proportion of variance (*β* = 1.00, 95% CI: −0.19 to 2.21, *P* = 0.09; Extended Data Fig. 1). Through iteratively introducing the correlated terms into the random slope model (exploring six further model structures), it is apparent that uncertainty increases most after the inclusion of spatial correlation (Extended Data Fig. 2).

## Predicting biodiversity change

Counterintuitively, accounting for correlative non-independence improves our capacity to make predictions ‘out of sample’—that is, for a withheld subset of data not used to develop the model—despite the large uncertainty at the level of the collective trend. Part of the value of these abundance trends is that they can be used to estimate which species and locations are likely to be declining or recovering, and when. To evaluate whether the correlated effect model improves our ability to make local-scale predictions, we tested each model’s ability to forecast new abundance observations and estimate population trends. For each dataset, we removed the final abundance observation in 50% of the population abundance time series and then evaluated each of the three models’ ability to predict this value. Next, we conducted leave-one-out cross-validation to assess trend prediction, removing a population time series (that is, trend) from each dataset and testing each model’s ability to recover this population’s abundance trend. We repeated this cross-validation 50 times for each of the 10 datasets. In each dataset, we report predictive accuracy for each of these approaches as the percentage error (PE), a metric describing the median of the absolute percentage difference between predicted and observed values; for example, with a 5% error, an abundance on the log scale of 1 would become 1.05. Summarizing across datasets, we report the mean and s.d.

Across the 10 datasets, the correlated effect model estimated the final abundance observation with 16.1% error (s.d. = 7.5%), 1.51 times more accurately than the random intercept model (mean = 24.4%, s.d. = 16.2%) and 1.13 times more accurately than the random slope model (mean = 18.3%; s.d. = 10.5%). The correlated effect model also performed best when estimating missing population trends, with an error of 18.3% (s.d. = 11.6%), 1.35 times more accurate than the random slope model (mean = 28.9%; s.d. = 25.5%). In one case, using the correlated effect model to capture the spatial, temporal and phylogenetic structures halved the trend prediction error, relative to the random slope model. The random slope model had a lower prediction error than the correlated effect model in only one dataset in the abundance assessment, and two datasets in the trend assessment.

**Fig. 3:**
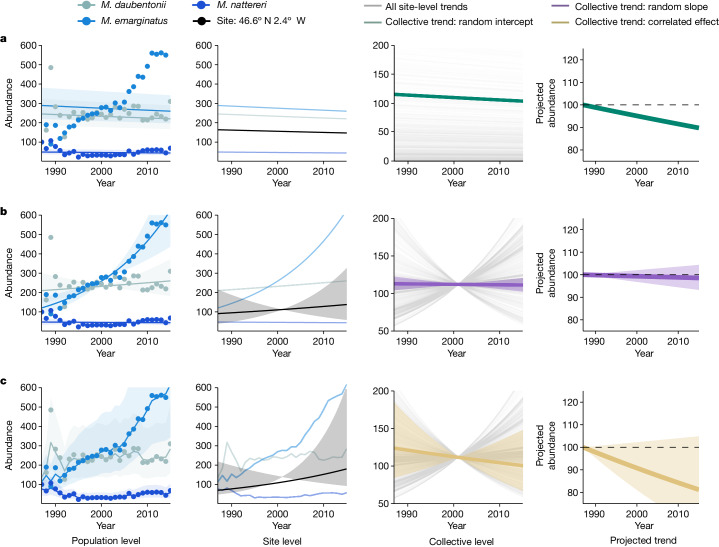
More complex models better represent population dynamics and improve the validity of conclusions across ecological scales. **a**–**c**, Example of how the three models (random intercept (**a**), random slope (**b**) and correlated effect (**c**)) describe abundance patterns at different ecological scales (finer ecological scales on the left). The population-level column showcases how each of the three models produce different estimates of abundance trends (lines are the median values with 95% credible interval shading) for all three bat species (genus *Myotis*) with data in a given location, with data points representing the observed abundance values. The site-level column depicts how the species’ trends, under different models, influence the site-level trend (that is, a trend for a given location; black), in which the line and 95% credible intervals describe the median trend and variability in trend (respectively) across all species in the given location. At the collective level, the median trend for each unique site is represented by a faded grey line, and the median collective trend coefficient and 95% credible intervals are depicted by the coloured line and shading. At the site and collective levels, credible intervals solely describe uncertainty in the main parameter of interest, the rate of change coefficient, not the intercept. The final column describes how a hypothetical population would change under the median collective trend coefficient and 50% credible intervals projected from a relative baseline abundance of 100. This example is based on data in the Living Planet. In each plot, we restrict the time frame to the temporal extent of the population-level trends (1987–2015), instead of the total temporal extent of our Living Planet sample. Source Data

The improved prediction in the correlated effect model is a consequence of handling temporal, spatial and phylogenetic non-independence. For the Living Planet data, recognizing temporal correlation between sequential abundance values (*ρ* = 0.52) introduces nonlinearity (residual variability in linear trends) in temporal trends, and more closely represents realistic population dynamics (Fig. 3, population level). Comparably, temporal non-independence in the Living Planet data was higher than the average across the ten datasets (*ρ* = 0.31, s.d. = 0.42, range: −0.65 to 0.99). Accounting for this temporal structure in population trends can also influence trend direction and uncertainty of species-level and site-level trends, relative to the random slope model (Fig. 3, site level). At the global level, the presence of temporal, spatial (proportion of variance captured by spatial correlation term = 0.30*)* and phylogenetic (proportion of variance captured by phylogenetic correlation term = 0.34) structures elevated the uncertainty around the overall trend (Extended Data Fig. 2), ultimately leading to more robust inference.

**Fig. 4:**
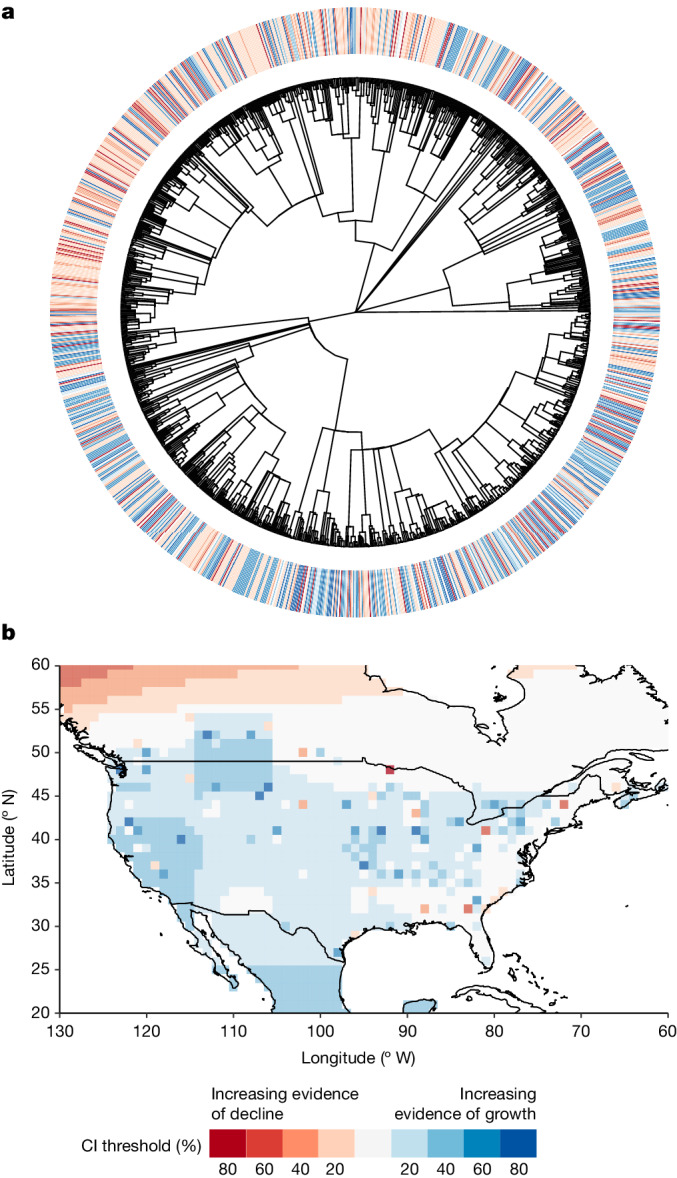
Abundance change varies over phylogenetic and spatial extents. Evidence of abundance change at different significance thresholds (for example, at an 80% CI threshold, dark red indicates evidence of declines whereas dark blue indicates evidence of increases). **a**, For the phylogeny, the species-level trends were derived by summing across hierarchical taxonomic random effects and phylogenetic correlation terms. Asymptotic species-level confidence thresholds were derived using uncertainty in phylogenetic predictions at multiple *z*-scores. To improve visualization, phylogenetic branch lengths are log transformed. **b**, For space, we take taxonomic and phylogenetic information from **a** for one iconic and abundant North American species, the American robin *Turdus migratorius*, and combine this with hierarchical and correlative spatial terms to make population-level predictions across terrestrial space. Asymptotic confidence thresholds were derived at the population scale (for example, species in a given site) using multiple *z*-scores. These predictions are drawn from the correlated effect model and BioTIME data (Supplementary Table 1). Source Data

The presence of the observed spatial and phylogenetic structures also has the added benefit of allowing us to make predictions beyond the species and location data (Fig. 4), offering important insight into species and locations most likely to exhibit declines and recoveries. Our ability to predict a given species trend is dependent on the species being contained and accurately described in a phylogeny. Efforts continue to expand the breadth and quality of phylogenetic information^28^, and across our 10 datasets, we were able to obtain phylogenetic information for 80% of species.

## Implications for biodiversity science

The abundance datasets we analyse are influential in policy, tracking progress towards biodiversity targets at national and international scales^16,17,29^, and so it is vital that any inference gained is both valid and reliable. Our work shows that when biodiversity change datasets are analysed without accounting for correlative non-independence among phylogeny, time and space, there is a substantial risk that trend uncertainty could be underestimated, trend direction misestimated and policy misinformed. Further, once uncertainty is appropriately attributed in the correlated effect model, we failed to detect a single significant trend in collective abundance across the 10 datasets under 95% credible intervals. This pervasive pattern points to a highly uncertain status in collective abundance trends; that is, it is unclear how biodiversity is changing across vast spatial and taxonomic scales once uncertainty is appropriately estimated.

The random intercept model, used by 43% of studies, underestimated trend uncertainty 26-fold. The random slope model, used by 50% of studies, performs better but still underestimates uncertainty 3.4-fold. This underestimated uncertainty has a substantial impact on trend inference, for which 9 datasets have significant trends at 50% credible intervals in the random intercept model compared to 7 datasets in the random slope model, and just 4 datasets in the correlated effect model. At 95% credible intervals, we found 8 significant collective trends in the random intercept model, 4 significant trends in the random slope model and zero significant trends in the correlated effect model. This raises questions about the robustness of existing estimates of abundance change in the literature.

Past estimates of abundance change have pointed to declines^15,21^, no net change^18,22,23^ and recovery^24^. This high variability across studies and datasets could be well founded given their different spatial, temporal and taxonomic scales, but it is also paradoxical given the expectation that biodiversity has declined under intense and widespread global change^1^. In the correlated effect model, we partially resolve this variability between datasets, as our results generalize under the common feature of substantial uncertainty. However, the absence of significant trends in the correlated effect model also further emphasizes the paradox of failing to detect biodiversity loss despite rapid global change. Ours is not the first study to fail to detect declining abundances. For instance, previous work has shown that most abundance trends exhibit no net change^23^, and that the magnitude of decline reduces after accounting for extreme values^18^ and random abundance fluctuations^19^. Other work suggests the current data collection infrastructure is too small and biased to detect a trend reliably^30^. Similarly, analyses of BioTIME suggest that declines are unlikely because environmental change generates winners as well as losers^2,22^, whose opposing population trends may cancel each other out at global scales.

All things considered, it seems likely that collective abundance trends over varied taxa exhibiting varied responses to varied degrees of environmental change may not present as significant even with vast quantities of data. Perhaps a more considered approach is necessary, focused on describing which taxonomic groups and specific locations are declining and recovering, and why. The correlated effect model is particularly well placed for exploring this question, as the integration of space and phylogeny allows us to explore the particular locations and clades for which abundance trends shift from stable to decline. Although the high uncertainty around collective trends limits our general understanding of abundance changes, we observe an increase in accuracy of abundance forecasts and trend predictions under the correlated effect model, delivering a much-needed improvement in prediction at local scales (Fig. 4). The more complex representation of space, time and phylogeny is key to this improved prediction, for which, as an example, a priori information on evolutionary history can help predict which species are likely to decline and recover. Our new methods offer the hope of greater clarity in biodiversity trend estimation across different datasets and geographies, to inform and guide adaptive conservation policy responses.

Despite failing to detect a decline in collective abundance across the ten datasets at 95% credible intervals, our results do not necessarily mean that wildlife abundances have not declined, or that the current estimates of trends are incorrect. It is possible that abundances may have increased on average, or perhaps declines have been far more extreme than we have previously imagined; simply, the uncertainty is too high to know. With this in mind, we re-emphasize calls to urgently expand data collection and improve trend detectability, but this will only help to estimate current and future biodiversity trends. Given the large uncertainty associated with our estimates of abundance change, it seems that past abundance patterns are lost and undetectable at present. A shift towards causal frameworks of detection and attribution is probably necessary to estimate past biodiversity change^14^.

In our study, we have solely focused on deriving the collective abundance trend given its potential political importance^17^, but the core statistical framework could be applied to other biodiversity data types (for example, occupancy data), adapted to other metrics (for example, species richness) and integrated into a global biodiversity observation system^14^. For instance, the species trend coefficients could be extracted from the model and used alongside estimates of absolute species abundance to determine the absolute change in populations^31^. Weightings could be included to increase the influence of common species, allowing us to reconcile and test for differences between collective abundance trends, biomass decline and individuals lost. Precise and accurate estimates of abundance change in time and space also underpin a variety of policy-relevant facets of biodiversity, including ecosystem function through abundance-weighted functional diversity^32,33^, energy flux, and population stability and resilience^34^.

The implications of our findings extend beyond biodiversity change. Spatial, temporal and phylogenetic data structures are common in ecological and evolutionary research. Under the presence of correlative non-independence, there is a potentially pervasive risk in the field that we have mis-specified our statistical models, violating data independence assumptions, and producing unreliable inference. However, this also presents an opportunity, as the correlative effect model could be adapted for a wide array of settings, improving inference across ecology and evolution. The model could also streamline workflows by simultaneously capturing spatial, phylogenetic and temporal structures, avoiding the need to capture terms in multiple separate analyses.

Our analytical advance offers new potential in predictive ecology, but given the severity of the potential implications of biodiversity loss^35^, it is vital that we continue to expand and improve these methods. We offer a general framework for addressing spatial, temporal and phylogenetic non-independence, but further advances are necessary, considering underlying issues around time-series length^36^, bias and non-probability^37^, nonlinearity and varied responses to environmental change^38^, modern data collection philosophies^39^ and rigorous analysis approaches^40^. This combination of improved methods and data has the potential to reveal patterns of biodiversity change and disentangle the complex processes shaping our ecosystems.

## Methods

### Data

We compiled ten datasets that describe population abundances through time^2–11,41^. These datasets represent some of the most influential in ecology and conservation biology, forming a basis for influential reports such as the Living Planet^15^, as well as a series of high-profile and highly cited publications (see Supplementary Table 2 for a full summary). For each dataset, we extracted the population abundance estimates, the accompanying time-stamps, the scientific names of the species, the name of the site (location) where the population was sampled and any site coordinates. For datasets to be included, they had to be open access, and contain multiple abundance time series for a selection of species and locations. Although these datasets are vital in biodiversity science, many of the datasets are prone to biases (for example, lacking tropical representation, and contain few plant and invertebrate species). The datasets have been compiled from a variety of methods, realms and systems, covering a vast array of spatial, taxonomic and temporal scales. Further, there is probably some overlap in data between datasets where population time series may occur in more than one dataset. We take no action to correct or acknowledge these biases and features, as our analysis is designed to show how model choice can have a substantial influence on inference in a variety of datasets, rather than to derive a consensus trend across datasets.

To account for correlative non-independence introduced by species’ shared evolutionary past, we extracted a phylogeny for each dataset. We used synthetic trees from the Open Tree of Life^42,43^ and estimated missing branch lengths using Grafen’s approach^44^ from the compute.brlen function in the R package ape^45^. The Open Tree of Life identified a phylogeny for 80% of species (*n* = 23,871); all other species were removed from the analysis. For studies with the overall aim of assessing biodiversity change, removing species could be problematic, as the collective trend would not be representative of all species. However, in our case, in which the aim is to assess how collective trend inference changes under a variety of modelling approaches, trimming the data to species with an accompanying phylogeny has no impact on our conclusions. Regardless, in sensitivity analyses in the Supplementary Information section entitled Phylogeny, we investigated this trade-off, and found that more than 1,000 species would have to be excluded from the data if higher-quality phylogenies were used (Supplementary Table 3). Further, inference is generally consistent across the datasets regardless of phylogeny quality (Supplementary Figs. 6 and 7).

After removing species not present in the Open Tree of Life topology, we further trimmed the data to include only higher-quality time series, removing the following: time series that contained zeros (which we considered extreme cases of extinctions or recolonizations) and time series with missing abundance values for a given year throughout the sampling duration (that is, we required consecutive abundance estimates). In all datasets except the two smallest ones—Atlantic reef fishes and Large carnivores—we further trimmed the datasets to keep only time series that had greater than or equal to the median number of abundance observations (that is, including the longest 50% of time series in each dataset). In some cases, this cutoff was not sufficient as the median number of observations in the time series equalled two. With only two abundance observations, trends are highly exposed to error purely driven by random fluctuations in abundance^19^. To partially address this issue, we imposed a further cutoff on these datasets, ensuring that each time series had at least five observations. These datasets are characterized in Supplementary Table 2. With our trimmed dataset, we derived a mean abundance in each year (in cases for which there were more than one observation per year) for each time series. In some datasets, there is a possibility that some species will have overlapping populations measured at different scales (for example, a national trend and a regional trend).

### Modelling

We explored which models have been used in the literature to infer abundance change. We focused on studies that characterized the average change in abundances over time, rather than studies assessing how many species are declining or increasing, as this avoids discretizing a numeric value; that is, we avoid having to define what change is necessary to be classified as a decline. To evaluate the diversity of approaches used to model abundance change over time in multi-species and/or multi-location datasets, we conducted a literature search in a selection of high-profile ecology journals over the past 13 years (Supplementary Information). Our search identified 282 research papers, 28 of which described approaches to model abundance change across multi-species and/or multi-location datasets. A further 16 methods were not detected in the literature search but were known priori to the authorship team, resulting in 44 different studies and/or methods. Models of abundance change varied in complexity, each containing their own assumptions, with no clear ‘standard’ approach for deriving the rate of change in abundance. However, across the 44 studies and/or methods that we compiled (Supplementary Table 1), five general approaches were present, as follows.

1. Abundance average: The simplest models derive an average or total abundance across all species or sites in a given year, and then regress average abundance against time. This approach fails to recognize any of the hierarchical structures in the data.
2. Trend average: A slightly more complex model, which estimates abundance change per population by fitting a series of log-linear modes of abundance against year; averaging over the extracted slope coefficients. This approach fails to propagate uncertainty in average rates of change of each population, and ignores the implicit spatial and taxonomic structures in the data, inducing pseudoreplication.
3. Random intercept: Some studies partially address the aforementioned pseudoreplication (for example, certain sites or species having multiple estimates) with mixed models, regressing log-linear abundance against year across all populations, while specifying that populations belong to a site and/or species. However, often this mixed model structure extends only to random intercepts, which only acknowledge that mean abundance can differ between sites, species and locations, and assumes that the abundance trends will all remain the same. This is a particularly common approach among the indicators from population monitoring schemes that shape policy^46^.
4. Random slope: In the scientific literature, it is common to use more complex models, with a similar structure to the random intercept model, but now also capturing the differences in abundance trends (not only mean abundances) across populations, sites and species with random slope terms.
5. Decomposition: This is the rarest of the approaches and deviates from the linear mixed model methods. Instead, the decomposition approach involves fitting generalized additive models through each time series to smooth abundance estimates and reduce noise. The smoothed time series is then decomposed into a time series of rates of change (or *λ* values), which are then averaged across species and biomes to derive estimates of the average change in abundance for each year across all of the time series.

The most common approaches were the random intercept and random slope models, used 19 and 23 times, respectively. The abundance average, trend average and decomposition approaches were rare, used just five, two and three times, respectively. Not all studies adopted just one approach, sometimes splitting their model into two steps (for example, using a random intercept model to estimate a given species trend across locations, which could then be aggregated across broader taxonomies with a random slope model). Further, all approaches regularly failed to account for temporal, spatial and phylogenetic structures (that is, closely related species are likely to have more similar trends than distant species), with only 14 of the 44 approaches accounting for temporal autocorrelation. Studies that accounted for phylogenetic or spatial covariance were comparably rarer—included in just six and three studies, respectively. Four studies attempted to account for two sources of correlative non-independence in their models, by first deriving population trends while accounting for temporal autocorrelation of abundances in time series, and then using phylogenetic least squares to aggregate these trends. However, no study captured more than one of these covariances simultaneously (for example, spatio-temporal models). Further, no study attempted to account for all three sources of correlative non-independence.

Given the apparent rarity of the abundance average, trend average and decomposition approaches in the literature, we focus on understanding how the dominant approaches (that is, the random intercept and random slope models) compare to our newly developed correlated effect model. Full model equations are available in the Supplementary Information.

#### Model 1 (random intercept)

In model 1, we fit a linear mixed-effect model between the natural logarithm of abundance and year, with five random intercepts: population (the unique time series), site (unique locations), region (broader spatial category to nest sites; flexibly determined on the basis of the spatial extent of the dataset), species (unique species) and genus (broader taxonomic category to nest species; measured as the parent node to the species tip). In the model, we do not specify any nesting of the population in the site and species random intercepts as the hierarchical structure of the data is poorly defined (for example, although populations always occur in a species and site, some species are nested in sites, and some sites are nested in species, creating a crossed random effect design). Model 1 assumes all populations, sites, regions, species and genera have the same trend in abundance.

#### Model 2 (random slope)

In model 2, we develop a linear mixed-effect model, in which we regress the natural logarithm of abundance against year, including population, site, region, species and genus all as random slopes. This builds on the random intercept model by allowing abundance–year slope coefficients to vary for each category in each random slope term (for example, each species can have a different slope)—not simply differing intercepts as in model 1. Unlike in model 1, we centre the year and abundances of each population time series at zero (for example, subtracting each year by the mean year in each population, and subtracting the log of each abundance by the mean log abundance value in each population). This centring fixes the *y* and *x* intercepts at zero for each slope, and is a convenient solution to account for variance captured by the random intercepts without increasing the number of parameters. To all intents and purposes, assuming that the objective is to characterize the abundance–year coefficient, the random slope model is equivalent to a model with random intercepts and slopes.

#### Model 3 (correlated effect)

Model 3 is structurally similar to model 2, but accounts for correlative non-independence. For temporal non-independence, we model the population level time series with a discrete first-order autoregressive temporal process, which assumes that sequential abundance observations in a time series will be more similar. To capture the spatial and phylogenetic correlative non-independence, we focus on non-independence across time-series trends (instead of abundance observations), assuming that trends in population abundances through time will be more similar in neighbouring sites and more closely related species. In models 1 and 2, we try to capture this non-independence with grouping categories (genus and region). However, in the correlated effect model, we more explicitly describe shared correlations between every species and site by specifying the covariance structures of our site and species random slopes. The site covariance matrix was derived by taking each site’s coordinates and estimating the pairwise Haversine (spherical) distance between the sites (for example, how far is every site from every other site). This was then converted into a matrix, normalized between 0 and 1, with values close to 1 indicating neighbouring sites, whereas values approaching 0 indicate distant sites. The species covariance matrix was derived by converting the phylogeny into a variance–covariance matrix, in which phylogenetic branch lengths describe the evolutionary distance between species.

All models were developed using Bayesian Integrated Nested Laplace Approximation (INLA)^47^ in R v.4.0.5 (ref. ^48^). We describe our model priors in the Supplementary Information section entitled Priors and validate our model assumptions in the Supplementary Information section entitled Assumptions (Supplementary Figs. 1–5). We also conduct additional sensitivity analyses exploring how phylogeny quality and how the addition of each correlative component (space, time or phylogeny) affect inference (see the Supplementary Information sections entitled Phylogeny and Component importance). We compiled the data using the following R packages: tidyverse^49^, countrycode^50^, janitor^51^, here^52^ and arrow^53^_._ Figures were produced using the following R packages: ggplot2^54^, ggtree^55^ and ape^45^.

### Outputs

#### Measuring non-independence

We assess whether correlative terms capture a meaningful proportion of variance in the data, by dividing the proportion of variance captured by the correlated slopes (for example, spatial covariance) by the combination of the variance captured by the correlated and independent slopes (for example, spatial covariance + site random slope + region random slope). This was carried out separately for the spatial and phylogenetic terms. As the spatial and phylogenetic components each contain three terms (an independent species or location slope, an independent genera or region slope and a correlated species or location slope), a proportional variance captured of 0.33 would indicate that the correlative slope captures an equal proportion of variance compared to the two independent slopes. A value greater than 0.33 indicates that correlative slopes account for more variation than independent random slopes. We measure temporal non-independence as the degree of correlation between sequential abundances (*ρ*).

#### Differing inference between the models

Using the mean and 50% credible intervals of the global trend (overall abundance–time coefficients), we display abundance projections for each model in each dataset. These projections are based on an arbitrary baseline abundance of 100, set at the first year of available data in each dataset, and this abundance would change according to the overall coefficients and credible intervals. For instance, with a 1% annual rate of change, an abundance in year zero of 100 would become 101 in year 1, and 164 in year 50. The purpose of these projections is to showcase varying abundance trajectories under different model complexities. We also report the fold change in the collective trend s.d. of the correlated effect model, relative to the random intercept and random slope models. This involved regressing fold change against category (for example, correlated effect versus random intercept) in a linear model. We report the mean fold change and 95% CIs. Model outputs are reported in Supplementary Tables 4 and 5.

#### Predictive performance

We assess the predictive performance of the different models by determining their ability to predict final observations in time series, and their ability to predict population trends of a given species in a given location. To test the predictive accuracy for the final observation in the time series, we removed the final observation from half of the time series in each dataset and predicted the missing values using each of the three models on the log scale. We report the percentage error (PE), a metric describing the median of the absolute percentage difference between predicted and observed values (for example, with a 5% error, an abundance on the log scale of 1 would become 1.05). This is calculated by finding the absolute difference between the true value and prediction, divided by the true value, before being multiplied by 100 and converted to an absolute error.

To test the accuracy of the population trend prediction, we conducted leave-one-out cross-validation, removing one population time series (or trend) from each dataset, and estimating the missing trend using the random slope and correlated effect models. We solely removed population time series with a trend not overlapping zero at 95% credible intervals (that is, populations changing significantly), to test our ability to identify which populations are changing or not. We repeated this process 50 times per dataset and compare the predicted trends to trends from a simplified correlated effect model, which contains a population-level slope and accounts for temporal autocorrelation, but does not include the spatial and phylogenetic correlation terms or any of the hierarchical terms, which have no influence on the required population-level inference. We measured trend-predictive performance using the same approaches as above (PE). In the random slope model, the population trend coefficients were derived by adding the species, location, genus, region and overall coefficients together, meaning that missing population trends can still be informed by other hierarchical information. For the correlated effect model, the population trend is informed by the phylogenetic and spatial variance–covariance matrices, as well as all hierarchical information in the random slope model. Prediction accuracy for each dataset is reported in Supplementary Tables 6 and 7.

#### Phylogenetic and spatial distribution of abundance change

To plot abundance change across a phylogeny, we derived species-level rates of change in abundance from the taxonomic (species and genera) and phylogenetic random effects. We incorporate uncertainty in species-level trend prediction by estimating the CI threshold by which a species would be considered to have increased or decreased. For instance, a negative trend at an 80% CI threshold would be considered stronger evidence of decline than a negative trend at a 20% interval threshold. We derive four asymptotic CI thresholds (20%, 40%, 60% and 80%) using the uncertainty (s.d.) from the phylogenetic random effect and a series of *z*-scores (0.25, 0.52, 0.84 and 1.28).

To plot abundance change across space, we focus solely on one abundant and iconic species, the American robin *T.* *migratorius*, as site-level trend variability is high at the community level (that is, community trends across space are rarely significant). To produce abundance change predictions for the American robin across space, we expanded the BioTIME spatial Haversine distance matrix (describing distances between each time series) by supplementing it with a gridded extent covering North America. This new grid had a latitudinal range of 20 to 60 and 1° spacing (for example, 15, 16 and so on), and longitudinal range of −130 to −60 with 1° spacing. This new matrix allows us to estimate expected covariance (similarity) in abundance trends for any pair of 1° cells across North America. We then derived the average rate of change in abundance across all hierarchical and correlative random effects, and used population-level trend uncertainty to derive the selection of CI thresholds described above.

### Reporting summary

Further information on research design is available in the Nature Portfolio Reporting Summary linked to this article.

## Online content

Any methods, additional references, Nature Portfolio reporting summaries, source data, extended data, supplementary information, acknowledgements, peer review information; details of author contributions and competing interests; and statements of data and code availability are available at 10.1038/s41586-024-07236-z.

### Supplementary information

Supplementary Information Supplementary text and data, Figs. 1–7 and Tables 1–7.

Reporting Summary

### Source data

Source Data Fig. 2

Source Data Fig. 3

Source Data Fig. 4

## Data Availability

All of the data used in the study are publicly available and accessible from the following links: RivFishTIME (10.1111/geb.13210), North American Breeding Birds (10.5066/P97WAZE5), BioTIME (10.1111/geb.12729), Living Planet (https://www.livingplanetindex.org/data_portal), CaPTrends (10.1111/geb.13587), ReSurveyGermany (10.25829/idiv.3514-0qsq70), UK Fish Counts (https://environment.data.gov.uk/dataset/ce2618db-d507-4671-bafe-840b930d2297), FishGlob (10.31219/osf.io/2bcjw), TimeFISH (10.1002/ecy.3966), Pilotto (https://zenodo.org/records/10638241). See comprehensive data descriptions in Supplementary Table 2 and data_compile.Rmd (https://zenodo.org/records/10638241). Source data are provided with this paper.

## Code availability

All code is available at https://zenodo.org/records/10638241.
