# Supplementary material for: Revealing uncertainty in the status of biodiversity change
Source: Nature. 2024 Mar 27;628(8009):788–94. doi: 10.1038/s41586-024-07236-z (PMC11041640; doi:10.1038/s41586-024-07236-z)
Supplement: Supplementary file 1 — Supplementary text and data, Figs. 1–7 and Tables 1–7. [file 41586_2024_7236_MOESM1_ESM.docx]

**Supplementary - Revealing uncertainty in the status of biodiversity change**

Model design

Prior to designing our models, we first explored what models have been used in the literature to explore abundance change patterns. We focussed on studies trying to characterise the average change in abundance over time, rather than studies attempting to assess how many species are declining or increasing, as this avoids discretizing a numeric value i.e. we avoid having to define what change is necessary to be classified as a ‘decline’.

To evaluate the diversity of approaches used to model abundance change over time in multi-species and/or multi-location datasets, we conducted a literature search within the Web of Science core collection. We searched for research papers containing the following boolean expression in their title, abstract or keywords: (insect OR plant OR vertebrate OR mammal OR bird OR fish OR reptile OR amphibian OR arthropod OR invertebrate) AND (population) AND (trend) AND (abundance OR density). This expression is unlikely to capture every single study describing abundance change across large taxonomic and spatial scales, but will provide a snapshot of common approaches. We constrained this search to only include modern approaches by limiting the temporal scale of the search to between 01-01-2010 and 01-03-2023. We also narrowed the search to only consider papers within established broad-scope and ecology journals: Nature, Nature communications, Nature Ecology and Evolution, Science, Science Advances, Ecology, PNAS, Journal of Ecology, Journal of Animal Ecology, Global Change Biology, Global Ecology and Biogeography, Journal of Biogeography.

Our search identified 282 research papers, which was updated to 281 after removing a retracted paper. Author TFJ read the abstract and title of each paper to identify works that were likely relevant (i.e. likely to have derived an average rate of change in abundance across a multi-species and/or multi-location dataset). The full list of research papers and accompanying abstracts is available in ‘https://zenodo.org/records/10638241’. For each paper deemed likely to contain relevant information (N = 55), TFJ read the paper in full, firstly checking if the authors had measured an average rate of change in abundance. In studies that had measured the average rate of change in abundance (N = 28), TFJ extracted the full reference, a text summary of the approach used to calculate the average rate of change, and whether the approach had attempted to account for spatial, phylogenetic or temporal correlative non-independence. Alongside the 28 relevant studies, TFJ also summarised the approaches of a further 16 (N = 44 total) methods not detected within the systematic search but were known a priori to the authorship team. A list of the 55 papers read in full is available in ‘full_read.csv’ within the code repository (https://zenodo.org/records/10638241).

Models of abundance change across multi-species multi-location datasets varied in complexity, each containing their own assumptions, with no clear ‘standard’ approach for deriving the rate of change in abundance. However, across the 44 studies/methods we compiled, five general approaches were present (Table S1):

1. Abundance average - The simplest models derive an average or total abundance across all species or sites in a given year, and then regress average abundance against time. This approach fails to recognise any of the hierarchical structure in the data. N = 5
2. Trend average - A slightly more complex model, which estimates abundance change per population by fitting a series of log-linear modes of abundance against year; averaging over the extracted slope coefficients. This approach fails to propagate uncertainty within average rates of change of each population, and ignores the implicit spatial and taxonomic structure within the data, inducing pseudoreplication. N = 2
3. Random intercept - Some studies partially address the aforementioned pseudoreplication (e.g. certain sites or species having multiple estimates) with mixed models, regressing log-linear abundance against year across all populations, whilst specifying that populations belong to a site and/or species. However, often this mixed model structure only extends to random intercepts , which only acknowledge that mean abundance can differ between sites, species and location, but assumes that the abundance trends will all remain the same. This is a particularly common approach amongst the indicators from population monitoring schemes which shape policy (e.g. the BTO bird monitoring scheme). N = 19
4. Random slope - In the scientific literature, it is common to use more complex models, with a similar structure to the Random intercept model, but now capturing the differences in abundance trends across populations, sites and species with random slope terms. N = 23
5. Decomposition - This is the rarest of the approaches and deviates from the linear mixed model approaches. Instead, the decomposition approach involves fitting generalised additive models (GAMs) through each time series to smooth abundance estimates and reduce noise. The smoothed time series is then decomposed into a timeseries of rates of change (or lambdas), which are then averaged across species and biomes to derive estimates of the average change in abundance for each year across all the time-series. N = 3

The most common approaches were the random intercept and random slope models, used 19 and 22 times, respectively. The abundance average, trend average and decomposition approaches were rare, used just 5, 2, and 3 times, respectively. Not all studies adopted just one approach, sometimes splitting their model into two steps e.g. using a random intercept model to estimate a given species trend across locations, which could then be aggregated across broader taxonomies with a random slope model. As a result, we detected 51 approaches across the 44 studies. Further, all approaches regularly failed to recognise that abundance patterns are shaped by implicit temporal, spatial and phylogenetic signals (i.e. closely related species are likely to have more similar trends than distant species), with only 14 (32%) of the 44 studies accounting for temporal autocorrelation. Phylogenetic and spatial covariance were comparably rarer - included in just 6 (14%) and 3 (7%) studies respectively. Four studies (9%) attempted to account for two sources of correlative non-independence within their models, by first deriving population trends whilst accounting for temporal autocorrelation of abundances within time series, and then using phylogenetic least squares to aggregate these trends. However, no study captured more than one of these covariances simultaneously (e.g. spatio-temporal models for instance). No study attempted to account for all three sources of correlative non-independence.

**Table S1.** Description of approach for deriving estimates of abundance change across multi-species and/or multi-location datasets across a variety of research articles and indicators. In each description we summarise the approach taken, what model it most closely matches too, and whether the studies accounted for underlying correlation structures. A selection of articles were identified outside of the systematic literature search, this information is noted in the description. Our descriptions purely represent an approximate summary of each model’s core structure, and so some of our model descriptions may be incomplete. As such, we do draw any direct comparison between any model in this table and our newly developed correlated effect mode

| **Reference** | **Description** |
| --- | --- |
| Barnes, M.D., Craigie, I.D., Harrison, L.B., Geldmann, J., Collen, B., Whitmee, S., Balmford, A., Burgess, N.D., Brooks, T., Hockings, M. and Woodley, S., 2016. Wildlife population trends in protected areas predicted by national socio-economic metrics and body size. *Nature communications*, *7*(1), p.12747. | Approach: Analysis is split into two stages 1) For each population, the log of abundance is regressed against year (continuous) to derive each population’s rate of change. 2) Rates of change are then regressed against covariates with species and site intercepts.  Model match: Functionally similar to the **random slope** model but split into two stages.  Correlation structures: No mention or assessment of any correlative non-independence. |
| Bongers, F., Ewango, C.E., van der Sande, M.T. and Poorter, L., 2020. Liana species decline in Congo basin contrasts with global patterns. *Ecology*, *101*(5), p.e03004. | Approach: Analysis is split into two stages 1) For each population, the log of abundance is regressed against year (continuous) to derive each population’s rate of change. 2) Rates of change are averaged to derive the mean growth rate.  Model match: Functionally similar to the **trend average** model but split into two stages.  Correlation structures: No mention or assessment of any correlative non-independence. |
| Bosch, J., Fernández‐Beaskoetxea, S., Garner, T.W. and Carrascal, L.M., 2018. Long‐term monitoring of an amphibian community after a climate change‐and infectious disease‐driven species extirpation. *Global change biology*, *24*(6), pp.2622-2632. | Approach: Analysis is split into two stages 1) Log abundance is regressed against lag-1 abundance in each time-series, which is somewhat equivalent to including an AR1 term. Model also includes a main effect of site (so mean abundance differs between sites) and year (as a factor, so mean abundance differs between years), resembling a random intercept model. This model is then run for each species. 2) Poll count comparison i.e. contrasting number of species declining/increasing based off the outputs from the random intercept model..  Model match: Functionally similar to the **random intercept** model.  Correlation structures: Accounts for temporal autocorrelation |
| Bowler, D.E., Hof, C., Haase, P., Kröncke, I., Schweiger, O., Adrian, R., Baert, L., Bauer, H.G., Blick, T., Brooker, R.W. and Dekoninck, W., 2017. Cross-realm assessment of climate change impacts on species’ abundance trends. *Nature ecology & evolution*, *1*(3), p.0067. | Approach: Analysis is split into two stages 1) Log abundance is regressed against year (factor) and site (factor) for each species. AR1 within time series is captured. 2) Species trends are averaged in a phylogenetic least squares  Model match: Analysis contains components of both the **random intercept** and **random slope**, acknowledging variable trends within species, but assuming species trends are uniform across sites.  Correlation structures: Accounts for temporal autocorrelation within time series. Then separately phylogenetic correlation across trends. |
| Crossley, M.S., Meier, A.R., Baldwin, E.M., Berry, L.L., Crenshaw, L.C., Hartman, G.L., Lagos-Kutz, D., Nichols, D.H., Patel, K., Varriano, S. and Snyder, W.E., 2020. No net insect abundance and diversity declines across US Long Term Ecological Research sites. *Nature Ecology & Evolution*, *4*(10), pp.1368-1376. | Approach: Analysis is split into two stages 1) For each population, the log of abundance is regressed against year (continuous) to derive each population’s rate of change. Accounts for AR1 structures. 2) Rates of change are averaged across populations to derive the global mean (tests with 1-sample T).  Model match: Functionally similar to the **trend average** model but split into two stages.  Correlation structures: Accounts for temporal autocorrelation within time series |
| Crossley, M.S., Smith, O.M., Berry, L.L., Phillips‐Cosio, R., Glassberg, J., Holman, K.M., Holmquest, J.G., Meier, A.R., Varriano, S.A., McClung, M.R. and Moran, M.D., 2021. Recent climate change is creating hotspots of butterfly increase and decline across North America. *Global Change Biology*, *27*(12), pp.2702-2714. | Approach: Analysis is split into two stages 1) Total abundance (across species) is calculated in each site and year. 2) Total abundance is regressed against year with spatially varying slopes  Model match: Analysis contains components of both the **abundance average** and **random slope**, acknowledging variable trends across space, but ignoring the different trends within species.  Correlation structures: Accounts for spatial structure in total abundance trends. |
| Daskalova, G.N., Myers-Smith, I.H. and Godlee, J.L., 2020. Rare and common vertebrates span a wide spectrum of population trends. *Nature communications*, *11*(1), p.4394. | Approach: Analysis is split into two stages 1) Annual rates of change are derived for each population using state space models. 2) Phylogenetic generalised linear models are used to look for covariate effects. Authors account for uncertainty in population trends.  Model match: Functionally similar to the **random slope** model but split into two stages.  Correlation structures: Accounts for temporal autocorrelation in populations, and phylogenetic correlation between slopes. |
| Falaschi, M., Manenti, R., Thuiller, W. and Ficetola, G.F., 2019. Continental‐scale determinants of population trends in European amphibians and reptiles. *Global Change Biology*, *25*(10), pp.3504-3515. | Approach: Analysis is split into two stages 1) Annual rates of change are derived for each population, in the form of Fisher Z effect sizes. 2) Linear mixed model, with trend as the response and spatial and taxonomic random effects  Model match: Functionally similar to the **random slope** model but split into two stages.  Correlation structures: Tested for spatial and phylogenetic signal in the residuals and found no effect. |
| Geppert, C., Perazza, G., Wilson, R.J., Bertolli, A., Prosser, F., Melchiori, G. and Marini, L., 2020. Consistent population declines but idiosyncratic range shifts in Alpine orchids under global change. *Nature communications*, *11*(1), p.5835. | Approach: Abundance regressed against year and covariates, with a poisson error term, and a species random intercept.  Model match: **Random intercept** model without spatial terms.  Correlation structures: No mention or assessment of any correlative non-independence. |
| He, F., Zarfl, C., Bremerich, V., David, J.N., Hogan, Z., Kalinkat, G., Tockner, K. and Jähnig, S.C., 2019. The global decline of freshwater megafauna. *Global Change Biology*, *25*(11), pp.3883-3892. | Approach: Abundance is regressed against year in the form of a generalised additive model (non-linear). Yearly rates of change are then derived, and averaged for each year e.g. the mean rate of change for each year.  Model match: The **decomposition** approach  Correlation structures: No mention or assessment of any correlative non-independence. |
| Jiguet, F., Gregory, R.D., Devictor, V., Green, R.E., Vorisek, P., Van Strien, A. and Couvet, D., 2010. Population trends of European common birds are predicted by characteristics of their climatic niche. *Global change biology*, *16*(2), pp.497-505. | Approach: Analysis is split into two stages: 1) Log abundance is regressed against lag-1 abundance in each time-series, which is somewhat equivalent to including an AR1 term. Model also includes a main effect of site (so mean abundance differs between sites) and year (as a factor, so mean abundance differs between years), resembling a random intercept model. This model is then run for each species. 2) Species trends are averaged in a phylogenetic least squares.  Model match: Analysis contains components of both the **random intercept** and **random slope**, acknowledging variable trends within species, but assuming species trends are uniform across sites.  Correlation structures: Accounts for temporal autocorrelation in time series, and phylogenetic correlations across species. |
| Jørgensen, P.S., Böhning‐Gaese, K., Thorup, K., Tøttrup, A.P., Chylarecki, P., Jiguet, F., Lehikoinen, A., Noble, D.G., Reif, J., Schmid, H. and van Turnhout, C., 2016. Continent‐scale global change attribution in European birds‐combining annual and decadal time scales. *Global Change Biology*, *22*(2), pp.530-543. | Approach: Analysis is split into two stages: 1) Log abundance is regressed against lag-1 abundance in each time-series, which is somewhat equivalent to including an AR1 term. Model also includes a main effect of site (so mean abundance differs between sites) and year (as a factor, so mean abundance differs between years), resembling a random intercept model. This model is then run for each species. 2) Linear mixed model, with trend as the response and spatial/ taxonomic random effects  Model match: Analysis contains components of both the **random intercept** and **random slope**, acknowledging variable trends within species, but assuming species trends are uniform across sites.  Correlation structures: Accounts for temporal autocorrelation within time-series. |
| van Langevelde, F., Braamburg‐Annegarn, M., Huigens, M.E., Groendijk, R., Poitevin, O., van Deijk, J.R., Ellis, W.N., van Grunsven, R.H., de Vos, R., Vos, R.A. and Franzén, M., 2018. Declines in moth populations stress the need for conserving dark nights. *Global change biology*, *24*(3), pp.925-932. | Approach: Analysis is split into two stages: 1) Abundances summed across different locations to derive species total abundance for each year. 2) Total abundance regressed against year whilst controlling for phylogeny. Species trends aggregated into covariate groups.  Model match: **Abundance average** followed by **random slope** model  Correlation structures: Accounts for phylogeny only. |
| Lehikoinen, A., Brotons, L., Calladine, J., Campedelli, T., Escandell, V., Flousek, J., Grueneberg, C., Haas, F., Harris, S., Herrando, S. and Husby, M., 2019. Declining population trends of European mountain birds. *Global Change Biology*, *25*(2), pp.577-588. | Approach: Analysis is split into two stages 1) Log abundance is regressed against lag-1 abundance in each time-series, which is somewhat equivalent to including an AR1 term. Model also includes a main effect of site (so mean abundance differs between sites) and year (as a factor, so mean abundance differs between years), resembling a random intercept model. This model is then run for each species. 2) Yearly rates of change are then extracted, and averaged for each year e.g. the mean rate of change for each year.  Model match: Functionally similar to the **random intercept** model but split into two stages.  Correlation structures: Accounts for temporal autocorrelation within time series. |
| Pollock, H.S., Toms, J.D., Tarwater, C.E., Benson, T.J., Karr, J.R. and Brawn, J.D., 2022. Long-term monitoring reveals widespread and severe declines of understory birds in a protected Neotropical forest. *Proceedings of the National Academy of Sciences*, *119*(16), p.e2108731119. | Approach: Analysis is split into two stages 1) Derived species trends in a couple of ways, but all with the same core principle, where abundance is a product of year (continuous), site and season i.e. the random intercept model. 2) Trends aggregated with a phylogenetic least squares meta regression, which allows uncertainty in the first modelling stage to be incorporated into the final model.  Model match: Analysis contains components of both the **random intercept** and **random slope**, acknowledging variable trends within species, but assuming species trends are uniform across sites.  Correlation structures: Tests for temporal autocorrelation within time series. Accounts for phylogenetic correlation between trends. |
| Reif, J. and Hanzelka, J., 2020. Continent‐wide gradients in open‐habitat insectivorous bird declines track spatial patterns in agricultural intensity across Europe. *Global Ecology and Biogeography*, *29*(11), pp.1988-2013. | Approach: Analysis is split into two stages: 1) Extracted national bird abundance trends from another study (Referenced as Birdlife 2015 but no functioning link; most likely refers to the PECBBMS TRIM indices https://pecbms.info/trends-and-indicators/species-trends/). 2) Then fits bird abundance trends with a GAMM containing latitude and longitude (so some recognition of spatial structure); but it's unclear how many knots were used etc (or whether this was just linear). Also contained species and family random effects, which the study states captures phylogenetic signal, but they never tested this.  Model match: Analysis contains components of both the **random intercept** and **random slope**, acknowledging variable trends within species, but assuming species trends are uniform across sites.  Correlation structures: Uses latitude and longitude in a spatial gamm to capture spatial structure. |
| Albaladejo‐Robles, G., Böhm, M. and Newbold, T., 2023. Species life‐history strategies affect population responses to temperature and land‐cover changes. *Global Change Biology*, *29*(1), pp.97-109. | Approach: Analysis is split into two stages: 1) For each population, the log of abundance is regressed against year (continuous) to derive each population’s rate of change. 2) Rates of change are then regressed against covariates with species and site intercepts.  Model match: Functionally similar to the **random slope** model but split into two stages.  Correlation structures: No mention or assessment of any correlative non-independence. |
| Spooner, F.E., Pearson, R.G. and Freeman, R., 2018. Rapid warming is associated with population decline among terrestrial birds and mammals globally. *Global change biology*, *24*(10), pp.4521-4531. | Approach: Analysis is split into two stages: 1) GAM used to smooth population time-series. Mean lambda then derived. 2) Mean lambda modelled against covariates with species and site as random effects  Model match: Functionally similar to the **random slope** model but split into two stages.  Correlation structures: No mention of assessment of any correlative non-independence. |
| Stephens, P.A., Mason, L.R., Green, R.E., Gregory, R.D., Sauer, J.R., Alison, J., Aunins, A., Brotons, L., Butchart, S.H., Campedelli, T. and Chodkiewicz, T., 2016. Consistent response of bird populations to climate change on two continents. *Science*, *352*(6281), pp.84-87. | Approach: Analysis is split into two stages: 1) For each population, the log of abundance is regressed against year (continuous) to derive each population’s rate of change. 2) Rates of change modelled against covariates with species and site as random effects  Model match: Functionally similar to the **random slope** model but split into two stages.  Correlation structures: No mention or assessment of any correlative non-independence. |
| Van Klink, R., Bowler, D.E., Gongalsky, K.B., Swengel, A.B., Gentile, A. and Chase, J.M., 2020. Meta-analysis reveals declines in terrestrial but increases in freshwater insect abundances. *Science*, *368*(6489), pp.417-420. | Approach: Model log abundance as a product of year, with location random intercept and slope. Account for AR1 in sites. No species or taxonomic random effects  Model match: **Random slope** model  Correlation structures: Accounts for temporal autocorrelation |
| Welti, E.A., Roeder, K.A., de Beurs, K.M., Joern, A. and Kaspari, M., 2020. Nutrient dilution and climate cycles underlie declines in a dominant insect herbivore. *Proceedings of the National Academy of Sciences*, *117*(13), pp.7271-7275. | Approach: Analysis is split into two stages 1) All species within a site are summed into total abundance per year. 2) Linear model of log abundance against year, with no mention of random effects for space or species.  Model match: Analysis contains components of both the **abundance average** and **random slope**, acknowledging variable trends across space, but ignoring the different trends within species.  Correlation structures: No mention or assessment of any correlative non-independence. |
| Williams, J.J., Freeman, R., Spooner, F. and Newbold, T., 2022. Vertebrate population trends are influenced by interactions between land use, climatic position, habitat loss and climate change. *Global change biology*, *28*(3), pp.797-815. | Approach: Analysis is split into two stages: 1) For each population, the log of abundance is regressed against year (continuous) to derive each population’s rate of change. 2) Rates of change modelled against covariates with species and site as random effects  Model match: Functionally similar to the **random slope** model but split into two stages.  Correlation structures: No mention or assessment of any correlative non-independence. |
| Meller, K., Vähätalo, A.V., Hokkanen, T., Rintala, J., Piha, M. and Lehikoinen, A., 2016. Interannual variation and long‐term trends in proportions of resident individuals in partially migratory birds. Journal of Animal Ecology, 85(2), pp.570-580. | Approach: Log abundance is regressed against lag-1 abundance in each time-series, which is somewhat equivalent to including an AR1 term. Model also includes a main effect of site (so mean abundance differs between sites) and year (as a factor, so mean abundance differs between years), resembling a random intercept model. This model is then run for each species. No aggregation across species  Model match. **Random intercept** model but ran separately for each species.  Correlation structures: Accounts for temporal autocorrelation. |
| Kim, H., McComb, B.C., Frey, S.J., Bell, D.M. and Betts, M.G., 2022. Forest microclimate and composition mediate long‐term trends of breeding bird populations. *Global Change Biology,* 28(21), pp.6180-6193. | Approach: Uses a dynamic N-mix, from Dali and Madsen, to estimate trends for each species whilst accounting for imperfect detection. Only derives species trends. No aggregation to assemblage or community trends. Appears as though there is lag 1 abundance term similar to AR1.  Model match: Species level trends are derived through a structure resembling a **Random intercept** model, where all sites have the same trend but different starting abundance  Correlation structures: Appears to account for temporal autocorrelation. |
| Hunter, M.D., Kozlov, M.V., Itämies, J., Pulliainen, E., Bäck, J., Kyrö, E.M. and Niemelä, P., 2014. Current temporal trends in moth abundance are counter to predicted effects of climate change in an assemblage of subarctic forest moths. *Global Change Biology*, 20(6), pp.1723-1737. | Approach: Analysis is split into two stages: 1) Abundances summed across different locations to derive species total abundance for each year. 2) Pearson correlation between log total abundance and year. Species trends never aggregated.  Model match. **Abundance average** model (i.e. assumes all sites have the same trend) but ran separately for each species.  Correlation structures: No mention or assessment of any correlative non-independence. |
| Schipper, A.M., Belmaker, J., de Miranda, M.D., Navarro, L.M., Böhning‐Gaese, K., Costello, M.J., Dornelas, M., Foppen, R., Hortal, J., Huijbregts, M.A. and Martín‐López, B., 2016. Contrasting changes in the abundance and diversity of North American bird assemblages from 1971 to 2010. *Global change biology,* 22(12), pp.3948-3959. | Approach: Analysis is split into two stages: 1) Abundance of assemblage is derived, meaning all species abundances are combined. 2) Study then assesses temporal trend per site/route; accounts for temporal autocorrelation within total abundance of assemblage with an AR1 GLS. Ignores phylogenetic structure as a consequence of simply adding abundances of all species.  Model match. **Abundance average** model (i.e. assumes all species have the same trend) and **Random slope** model  Correlation structures: Accounts for temporal autocorrelation |
| Genner, M.J., Sims, D.W., Southward, A.J., Budd, G.C., Masterson, P., Mchugh, M., Rendle, P., Southall, E.J., Wearmouth, V.J. and Hawkins, S.J., 2010. Body size‐dependent responses of a marine fish assemblage to climate change and fishing over a century‐long scale. *Global Change Biology*, 16(2), pp.517-527. | Approach: Takes the mean abundance across all sites and species in a size class for each year. The summed yearly abundance is then regressed against time with a pearson correlation  Model match: **Abundance average** (i.e. assumes all sites and species have the same trend) but ran separately for each species.  Correlation structures: No mention or assessment of any correlative non-independence. |
| Schowalter, T.D., Pandey, M., Presley, S.J., Willig, M.R. and Zimmerman, J.K., 2021. Arthropods are not declining but are responsive to disturbance in the Luquillo Experimental Forest, Puerto Rico. *Proceedings of the National Academy of Sciences*, 118(2), p.e2002556117. | Approach: Model negative binomal of density against year, with random effect (looks like intercept)) of location. Each species is modelled separately and not aggregated:  Model match: Functionally similar to **Random intercept**  Correlation structures: No mention or assessment of any correlative non-independence. |
| BTO, Breeding Bird Survey (2020), (available at https://www.bto.org/our-science/publications/birdtrends/2020/methods/breeding-bird-survey). | Approach: Analysis is split into two stages. 1) For each species, a poisson generalised linear model is run where abundance is predicted by two categorical fixed effects of site and year. This assumes all sites have the same linear trend but varying intercept, and that abundance observations at neighbouring points in time are independent. 2) To produce national averages, the mean predicted abundance estimates (average across all sites) for each species are derived, and then abundance estimates across species are smoothed i.e., not acknowledging the variable trends in species, and simply following the general pattern in abundances  Model match: Analysis most closely resembles the **random intercept** model  Correlation structures: No mention or assessment of any correlative non-independence.  Notes: This approach was not found through the systematic literature search. Used on the UK Breeding Bird Survey and Common Bird Survey data and indicators. |
| R. M. Fewster, S. T. Buckland, G. M. Siriwardena, S. R. Baillie, J. D. Wilson, Analysis of Population Trends for Farmland Birds Using Generalized Additive Models. Ecology. 81, 1970–1984 (2000). | Approach: Analysis is split into two stages 1) For each species, a poisson generalised additive model is run where abundance is predicted by a categorical fixed effects of site and a smoothing effect of year. This assumes all sites have the same smoothed (likely non-linear) trend. 2) To produce national averages, the mean predicted abundance estimates (average across all sites) for each species are derived, and then abundance estimates across species are smoothed i.e., not acknowledging the variable trends in species, and simply following the general pattern in abundances  Model match: Analysis most closely resembles the **random intercept** model but does acknowledge non-linear abundance patterns  Correlation structures: No mention or assessment of any correlative non-independence.  Notes: This approach was not found through the systematic literature search. Used on the UK Breeding Bird Survey and Common Bird Survey data and indicators. |
| A. C. Smith, B. P. M. Edwards, North American Breeding Bird Survey status and trend estimates to inform a wide range of conservation needs, using a flexible Bayesian hierarchical generalized additive model. Ornithol. Appl. 123, duaa065 (2021). | Approach: For each species, a poisson generalised additive model is run where abundance is predicted by a random slope of site interacting with a smoothing effect of year. This allows sites to have varying smoothed (likely non-linear) trends. Focus is on the species level trends. Approach to aggregate trends is not described  Model match: Species and sites are allowed to have varying trends as in the **random slope** model. Site level trends are smooth, not linear. No trend aggregation described  Correlation structures: No mention or assessment of any correlative non-independence.  Notes: This approach was not found through the systematic literature search. Used on the North American breeding bird survey data. |
| A. C. Smith, B. P. M. Edwards, North American Breeding Bird Survey status and trend estimates to inform a wide range of conservation needs, using a flexible Bayesian hierarchical generalized additive model. Ornithol. Appl. 123, duaa065 (2021). | Approach: For each species, a poisson generalised additive model is run where abundance is predicted by a random slope of site interacting with a smoothing effect of year. Unlike the above model, this also includes an additional random term to account for site-level random deviations from the smooth. This allows sites to have varying smoothed (likely non-linear) trends. Focus is on the species level trends. Approach to aggregate trends is not described  Model match: Species and sites are allowed to have varying trends as in the **random slope** model. Site level trends are smooth, not linear. No trend aggregation described  Correlation structures: No mention or assessment of any correlative non-independence.  Notes: This approach was not found through the systematic literature search. Used on the North American breeding bird survey data. |
| J. R. Sauer, K. L. Pardieck, D. J. Ziolkowski, A. C. Smith, M.-A. R. Hudson, V. Rodriguez, H. Berlanga, D. K. Niven, W. A. Link, The first 50 years of the North American Breeding Bird Survey. The Condor. 119, 576–593 (2017). | Approach: For each species, a poisson generalised linear model is run where abundance is predicted by a random slope of site interacting with a linear effect of year. This model also includes an additional random term *c* to account for site-level random intercepts. This allows sites to have varying log-linear trends. Focus is on the species level trends. Approach to aggregate trends is not described  Model match: Species and sites are allowed to have varying trends like the **random slope** model. Site level trends are linear. No trend aggregation described.  Correlation structures: No mention or assessment of any correlative non-independence.  Notes: This approach was not found through the systematic literature search. Used on the North American breeding bird survey data. |
| J. E. Houlahan, C. S. Findlay, B. R. Schmidt, A. H. Meyer, S. L. Kuzmin, Quantitative evidence for global amphibian population declines. Nature. 404, 752–755 (2000). | Approach: Study calculates yearly lambdas (Nt/Nt+1) for each pair of abundance observations in every population time series, and then averages lambdas across all populations in each year to report the estimated mean lambda/year. Study also uses a second method, calculating the proportion of populations increasing vs decreasing with kendall tau correlation between abundance and time  Model match: Functionally similar to the **decomposition** model but split into two stages.  Correlation structures: No mention or assessment of any correlative non-independence.  Notes: This approach was not found through the systematic literature search. |
| C. S. Robbins, J. R. Sauer, R. S. Greenberg, S. Droege, Population declines in North American birds that migrate to the neotropics. Proc. Natl. Acad. Sci. 86, 7658–7662 (1989). | Approach: Analysis is split into two stages 1) For each population, the log of abundance is regressed against year (continuous) to derive each population’s rate of change. 2) Rates of change are then regressed against covariates with species and site intercepts.  Model match: Functionally similar to the **random slope** model but split into two stages.  Correlation structures: No mention or assessment of any correlative non-independence.  Notes: This approach was not found through the systematic literature search. |
| J. A. Hutchings, C. Minto, D. Ricard, J. K. Baum, O. P. Jensen, Trends in the abundance of marine fishes. Can. J. Fish. Aquat. Sci. 67, 1205–1210 (2010). | Approach: The natural log of abundance (biomass) is regressed against year, with a random intercept term for each population. Model also captures temporal autocorrelation with an auto-regressive 1 process to indicate correlation between abundance neighbouring observations  Model match: **Random intercep**t model. Fails to acknowledge species pseudoreplication.  Correlation structure: Accounts for temporal autocorrelation  Notes: This approach was not found through the systematic literature search. |
| N. Poulet, L. Beaulaton, S. Dembski, Time trends in fish populations in metropolitan France: insights from national monitoring data. J. Fish Biol. 79, 1436–1452 (2011). | Approach: For each species, a poisson generalised linear model is run, with abundance as the response, regressed against year (treated continuously) and site (as a factor). This assumes abundance-time trends are the same across all sites, but acknowledges that species will have varying trends. There is no information on how trends are aggregated at the national/global-level.  Model match: Analysis contains components of both the **random intercept** and **random slope**, acknowledging variable trends across species, but ignoring the different trends across space.  Correlation structures: No mention or assessment of any correlative non-independence.  Notes: This approach was not found through the systematic literature search. |
| S. Seibold, M. M. Gossner, N. K. Simons, N. Blüthgen, J. Müller, D. Ambarlı, C. Ammer, J. Bauhus, M. Fischer, J. C. Habel, K. E. Linsenmair, T. Nauss, C. Penone, D. Prati, P. Schall, E.-D. Schulze, J. Vogt, S. Wöllauer, W. W. Weisser, Arthropod decline in grasslands and forests is associated with landscape-level drivers. Nature. 574, 671–674 (2019). | Approach: A poisson generalised linear model is run, with abundance as the response, regressed against year (treated continuously), with site and region as nested random intercepts. This assumes abundance-time trends are the same across all sites, but further, as all species are included in the one model, each site-level intercept represents multiple species. Species trends are ignored  Model match: **Random intercept** model, but without taxonomic structure  Correlation structures: No mention or assessment of any correlative non-independence.  Notes: This approach was not found through the systematic literature search. |
| G. N. Daskalova, A. B. Phillimore, I. H. Myers-Smith, Accounting for year effects and sampling error in temporal analyses of invertebrate population and biodiversity change: a comment on Seibold et al. 2019. Insect Conserv. Divers. 14, 149–154 (2021). | Approach: A poisson generalised linear model is run, with abundance as the response, regressed against year (treated continuously), with site and region as nested random intercepts. There is also an additional random intercept of year, so year as treated as both a fixed and random effect. This model assumes abundance-time trends are the same across all sites, but further, as all species are included in the one model, each site-level intercept represents multiple species. Species trends are ignored  Model match: **Random intercept** model, but without taxonomic structure  Correlation structures: No mention or assessment of any correlative non-independence.  Notes: This approach was not found through the systematic literature search. |
| G. N. Daskalova, A. B. Phillimore, I. H. Myers-Smith, Accounting for year effects and sampling error in temporal analyses of invertebrate population and biodiversity change: a comment on Seibold et al. 2019. Insect Conserv. Divers. 14, 149–154 (2021). | Approach: A poisson generalised linear model is run, with abundance as the response, regressed against year (treated continuously). The model includes correlated random slopes of abundance varying by year differently in sites and regions. This model assumes abundance-time trends differ across sites. Further, as all species are included in the one model, each site-level intercept and slope represents multiple species. Species trends are ignored  Model match: **Random intercept** model, but without taxonomic structure  Correlation structures: No mention or assessment of any correlative non-independence.  Notes: This approach was not found through the systematic literature search. |
| M. Dornelas, N. J. Gotelli, H. Shimadzu, F. Moyes, A. E. Magurran, B. J. McGill, A balance of winners and losers in the Anthropocene. Ecol. Lett. (2019), doi:10.1111/ele.13242. | Approach:Analysis is split into two stages 1) Study derives mean rate of change (lambda) per population time series by regressing the natural logarithm of abundance against the continuous variable year. 2) Trends (rates of change) are then aggregated to species/site level estimates in a further mixed model  Model match: Most closely resembles the **random slope** model, but instead of calculating rates of change within the model (as in our random slope model), rates of change are estimated in a preliminary step.  Correlation structures: No mention or assessment of any correlative non-independence.  Notes: This approach was not found through the systematic literature search. Used on the BioTIME dataset |
| F. Pilotto, I. Kühn, R. Adrian, R. Alber, A. Alignier, C. Andrews, J. Bäck, L. Barbaro, D. Beaumont, N. Beenaerts, S. Benham, D. S. Boukal, V. Bretagnolle, E. Camatti, R. Canullo, P. G. et al., Meta-analysis of multidecadal biodiversity trends in Europe. Nat. Commun. 11, 3486 (2020). | Approach: 1) Study derives estimated rate of change per population time series from a mann-kendall correlation between abundance and time. 2) Trends (rates of change) are then aggregates to species/site level estimates in a further meta regression which propagates uncertainty from stage 1 into the final aggregate  Model match: Most closely resembles the **random slope** model, but instead of calculating rates of change within the model (as in our random slope model), rates of change are estimated in a preliminary step.  Correlation structures: No mention or assessment of any correlative non-independence.  Notes: This approach was not found through the systematic literature search. Used on the European insects dataset |
| PECBMS, Production of national indices and trends (2022), (available at https://pecbms.info/methods/pecbms-methods/1-national-species-indices-and-trends/1-2-production-of-national-indices-and-trends/). | Approach: Analysis is split into two stages 1) Log abundance is regressed against lag-1 abundance in each time-series, which is somewhat equivalent to including an AR1 term. Model also includes a main effect of site (so mean abundance differs between sites) and year (as a factor, so mean abundance differs between years), resembling a random intercept model. This model is then run for each species. 2) Approach for aggregating to national-level and global-level is unclear.  Model match. **Random intercept** model but ran separately for each species.  Correlation structures: Accounts for temporal autocorrelation.  Notes: This approach was not found through the systematic literature search. Used on the PECBMS dataset |
| L. McRae, S. Deinet, R. Freeman, The Diversity-Weighted Living Planet Index: Controlling for Taxonomic Bias in a Global Biodiversity Indicator. PLOS ONE. 12, e0169156 (2017). | Approach: Analysis conducted in two stages 1) Each abundance time series is smoothed with a generalised additive model, to produce predicted values of abundance. Yearly pairwise-lambdas are then derived from these predicted values of abundance, essentially decomposing abundance time series into rate change (lambda) time series. 2) Lambdas are averaged each year at the species-level. Species level lambdas are then averaged to produce a global level estimate.  Model match: **Decomposition** model  Correlation structures: No mention or assessment of any correlative non-independence.  Notes: This approach was not found through the systematic literature search. Used on a sample of the Living Planet Data |

Given the apparent rarity of the trend average, simple average and decomposition approaches within the literature, we focus on understanding how the dominant approaches (i.e. the random intercept and random slope models) compare to our newly developed correlated effect model.

*Model 1. Random intercept*

In model 1, we fit a linear mixed effect model between the natural logarithm of abundance and year, with five random intercepts: population (the unique time series), site (unique locations), region (broader spatial category to nest sites; measured as the continent or ocean the site occurs in), species (unique species), and genus (broader taxonomic category to nest species; measured as the parent node to the species tip). Within the model, we do not specify any nesting between the site and species random intercepts as the hierarchical structure of the data is poorly defined e.g., whilst populations always occur within a species and site, some species are nested in sites, and some sites are nested in species, creating a crossed random effect design. Model 1 assumes all populations, sites, regions, species, and genera have the same trend in abundance.

*u_ijklm_ =* $u_{i}^{S}$ *+* $u_{j}^{L}$ *+* $u_{k}^{P}$ *+* $u_{l}^{G}$ *+* $u_{m}^{R}$

***u^S^*** *~ N(0,*$\sigma^{2}$*_S_I)*

***u^L^*** *~ N(0,*$\sigma^{2}$*_L_I)*

***u^P^*** *~ N(0,*$\sigma^{2}$*_P_I)*

***u^G^*** *~ N(0,*$\sigma^{2}$*_G_I)*

***u^R^*** *~ N(0,*$\sigma^{2}$*_R_I)*

*ȳ_ijklmt_ = b +* [$\beta$]*x_ijklmt +_ + u_ijklm_*

*y_ijklm_ N*(*ȳ_ijklmt_,*$\sigma^{2}$*_E_I*)

Where *u* represents the independent random intercept terms for species (*S*, index *i*), locations (*L*, index *j*), populations (*P*, index *k*), genera (*G*, index *l*) and regions (*R*, index *m*), all following a gaussian normal-distribution, with each varying according to their respective sigma hyperprior. These random intercepts vary around the overall model intercept (*b*), with a slope coefficient of $\beta$ describing abundance change over years (*x*). This formula describes expected abundances (*ȳ*) for each intercept grouping, at time point *t* (indexing of each abundance observation). *y* represents a vector of abundances for each population (index *k*), drawn from a gaussian normal-distribution with a mean *ȳ* and a residual error of $\sigma^{2}$*_E_*. *I* describes the identity matrix of the error terms.

*Model 2. Random slope*

In model 2, we develop a linear mixed effect model, where we regress the natural logarithm of abundance against year, including population, site, region, species, and genus all as random slopes. This builds on the random intercept model by allowing abundance-year slope coefficients to vary for each category in each random slope term (e.g., each species can have a different slope) - not simply differing intercepts as in model 1. Unlike model 1, we centre the year and abundances of each population time series at zero e.g. subtracting each year by the mean year in each population, and subtracting the log of each abundance value by the mean log abundance value in each population. This centering fixes the y and x intercepts at zero for each slope, and is a convenient solution to remove variance captured by the random intercepts without increasing the number of parameters.

*u_ijklm_ =* $u_{i}^{S}$ *+* $u_{j}^{L}$ *+* $u_{k}^{P}$ *+* $u_{l}^{G}$ *+* $u_{m}^{R}$

***u^S^*** *~ N(0,*$\sigma^{2}$*_S_I)*

***u^L^*** *~ N(0,*$\sigma^{2}$*_L_I)*

***u^P^*** *~ N(0,*$\sigma^{2}$*_P_I)*

***u^G^*** *~ N(0,*$\sigma^{2}$*_G_I)*

***u^R^*** *~ N(0,*$\sigma^{2}$*_R_I)*

*ȳ_ijklmt_ = b +* [$\beta$ *+ u_ijklm_*]*x_ijklmt_*

*y_ijklm_ N*(*ȳ_ijklmt_,*$\sigma^{2}$*_E_I*)

Where *u* represents the independent random slope terms for species (*S*, index *i*), locations (*L*, index *j*), populations (*P*, index *k*), genera (*G*, index *l*) and regions (*R*, index *m*), all following a gaussian normal-distribution, with each varying according to their respective sigma hyperprior. These independent random slopes vary around the overall slope coefficient of $\beta$ describing abundance change over years (*x*) - meaning the abundance-time slope coefficient is allowed to vary in each species, location, population, genera, and region. *b* describes the overall model intercept, which is included to support model convergence, but has a value of c.0 given the centering of the abundance and year values described above. This formula describes expected abundances (*ȳ*) for each slope grouping term, at time point *t* (indexing of each abundance observation). *y* represents a vector of log abundances for each population (index *k*), drawn from a gaussian normal-distribution with a mean *ȳ* and a residual error of $\sigma^{2}$*_E_*. *I* describes the identity matrix of the error terms.

*Model 3. Correlated effect*

Model 3 is structurally similar to model 2, but accounts for correlative non-independence structures. For temporal non-independence, we model the population level time series with a discrete autoregressive-1 (ar1) temporal process, which assumes neighbouring abundance observations within a time series will be more similar. To capture the spatial and phylogenetic correlative non-independence, we focus on non-independence across time series trends (instead of abundance observations), assuming trends in population abundances through time will be more similar in neighbouring sites and more closely related species. In model 1 and 2, we try to capture this non-independence with grouping categories (genus and region). However, in the correlated effect model, we more explicitly describe shared correlations between every species and site by specifying the covariance structure of our site and species random slopes. The site covariance matrix was derived by developing a matrix that describes the Haversine (spherical) distance between each site. To increase run time in the largest datasets (those with more than 250,000 observations), we rounded site coordinates to the nearest integer i.e. a latitude of 10.65 was set to 11. We normalised this spatial matrix between 0 and 1, with values close to 1 indicating neighbouring sites, whilst values approaching 0 indicate distant sites. The species covariance matrix was derived by extracting the variance-covariance matrix directly from the species’ phylogeny.

*u_ijklm_ =* $u_{i}^{S}$ *+* $u_{j}^{L}$ *+* $u_{k}^{P}$ *+* $u_{l}^{G}$ *+* $u_{m}^{R}$

***u^S^*** *~ N(0,*$\sigma^{2}$*_S_I)*

***u^L^*** *~ N(0,*$\sigma^{2}$*_L_I)*

***u^P^*** *~ N(0,*$\sigma^{2}$*_P_I)*

***u^G^*** *~ N(0,*$\sigma^{2}$*_G_I)*

***u^R^*** *~ N(0,*$\sigma^{2}$*_R_I)*

*v_ij_ =* $v_{i}^{S}$ *+* $v_{j}^{L}$

***v^S^*** *~ N(0,*$\sigma^{2}$*_S_Ω)*

***v^L^*** *~ N(0,*$\sigma^{2}$*_L_Δ)*

*ȳ_ijkt_ = b +* [$\beta$ *+ u_ijklm_ + v_ij_*]*x_ijkt_*

*y_ijk_ N*(*ȳ_ijkt_,*$\sigma^{2}$*_E_I +* $\sigma^{2}$*_A_𝛳*)

Where *u* represents the independent random slope terms for species (*S*, index *i*), locations (*L*, index *j*), populations (*P*, index *k*), genera (*G*, index *l*) and regions (*R*, index *m*), whilst *v* represents the correlated random slope terms for species (*S*, index *i*) and locations (*L*, index *j*). All random slopes, independent and correlated, follow a gaussian normal-distribution, with each varying according to their respective sigma hyperprior. However, the independent and correlated slopes differ, as *u* varies according to the identity matrix *I*, whilst *v* varies according to variance-covariances *Ω* and *Δ* which specify that covariance is present in neighbouring sites and more closely related species. These independent and correlated random slopes vary around the overall slope coefficient of $\beta$ describing abundance change over years (*x*) - meaning the abundance-time slope coefficient is allowed to vary in each species, location, and population. *b* describes the overall model intercept. This formula describes expected abundances (*ȳ*) for each slope grouping term, at time point *t* (indexing of each abundance observation). *y* represents a vector of abundances for each population (index *k*), drawn from a gaussian normal-distribution with a mean of *ȳ*. However, unlike model 2, the error term of this distribution has two components, the residual error of $\sigma^{2}$*_E_* as in model 2, and a new error term $\sigma^{2}$*_A_𝛳* which captures temporal non-independence by parameterising the correlation between neighbouring abundance values (*𝛳,* often called *rho*) and the left-over error from this process ($\sigma^{2}$*_A_*). Within this model, additional parameterisation options are available and could be explored in future work. For instance, to support analysis of time-series in continuous space (i.e. uneven temporal gaps in time series) users could embed an Ornstein–Uhlenbeck process ^56^. Further, we specify that all time-series, and in turn species, share the same autocorrelation parameter (*rho*) which may not be a fair assumption and could be allowed to vary between species. For space, users could consider the computationally more efficient spatial matern covariance ^57^ and allow spatial patterns to vary between species. For phylogeny, users could explore alternative evolutionary models ^58^.

Priors

Across all three models, we set normal priors on the fixed effects (*b -* $\beta$), centred at zero, with a variance of 1000 for the intercept, and 1 for the slope coefficient. These priors are vague, allowing the overall model intercept to vary between approximately -100 and 100 (i.e. substantial flexibility on the log-scale) and the overall slope to vary between approximately -2 and 2 (i.e. the overall population can increase/decrease 7-fold annualy). All random intercepts and slopes were assigned a normal prior, centred at zero, with an improper uniform hyperprior determining the standard deviation of normal priors. We used a uniform hyperprior following established recommendations ^59^. In the correlated effect model, we used a penalised complexity prior for the temporal ar-1 term. These penalised complexity priors are a principled approach that penalise departures from a base model based on probability statements ^60^. This is an intuitive and recommended prior for ar1 terms ^61^.

*Prob (*$\sigma$*_A_ >* $\sigma$*_0_) = a_0_,* $\sigma$*_0_ > 0, 0 < a_0_ < 1*

Where *Prob* describes the probability *a_0_* of the standard deviation captured by the ar1 term ($\sigma$*_A_)* exceeds a given threshold ($\sigma$*_0_*). Here, following published recommendations ^62^ and previous applications ^63^, we specify a threshold ($\sigma$*_0_*) three times larger than residual standard deviation in the random slope model, and set a probability (*a_0_*) of 0.01. Put simply, the probability of the standard deviation captured by the ar1 term exceeding three times the residual standard deviation is extremely low (*a_0_* = 0.01). This is analogous to a weakly informed prior. Alongside specifying the prior for the ar1 standard deviation, it's also important to set a prior of expected correlation (*𝛳; rho*) between abundance observations, under a similar probability statement.

*Prob (𝛳 >* $\sigma$*_1_) = a_1_, -1 <* $\sigma$*_1_ < 1,* $\sqrt{\frac{1 - \sigma1}{2}}$ *< a_1_ < 1*

Where *a_1_* describes the probability of the correlation (*𝛳*) between abundance values exceeding $\sigma$*_1_*. We specify a correlation threshold ($\sigma$*_1_*) of 0, and probability (*a_1_*) of 0.9 i.e. there is a high probability of observing at least some autocorrelation within the abundance time-series. This is the recommended approach in previous work ^64^. All models were run in INLA ^47^. INLA is very computationally efficient and all models can be run using a modest computer with 16GB of RAM. The largest datasets (e.g. BioTIME) take approximately one hour to complete.

Data

**Table S2.** Dataset name and description for each of our 10 datasets. Descriptions include temporal, taxonomic and spatial summaries.

| Dataset | Description |
| --- | --- |
| A: BioTIME ^2^ | Population abundance time series from the BioTIME dataset - representing all core taxa and realms. Covering 12,065 abundance time series, derived from 243,993 abundance observations. These time series represent 438 unique sites and 1,233 species. Our BioTIME sample is the product of the follow datasets ^3,65–78,78–129^.  Temporal extent: 1933-2018  Latitude extent: -77.6 - 67.8  Longitude extent: -179.8 - 179.2 |
| B: Living Planet ^8^ | Global population abundance time series for vertebrates. Covering 3,613 abundance time series, derived from 77,773 abundance observations. These time series represent 1,244 unique sites and 1,333 species.  Temporal extent: 1950 - 2020  Latitude extent: -77.8 - 78.9  Longitude extent: -179.6 - 180 |
| C: North American Breeding Birds ^3^ | Population abundance time series from the North American breeding bird survey. Covering 8,718 abundance time series, derived from 164,317 abundance observations. These time series represent 584 unique sites and 361 species.  Temporal extent: 1966 - 2019  Latitude extent: 25.9 - 67.0  Longitude extent: -165.3 - -55.4 |
| D: FishGlob ^9^ | Population abundance time series from the FishGlob database, describing abundances from the bottom-trawl survey for marine fishes. Covering 2,286 abundance time series, derived from 67,908 abundance observations. These time series represent 229 unique sites and 152 species.  Temporal extent: 1977 - 2020  Latitude extent: 26 - 62  Longitude extent: -178 - 21 |
| E: RivFishTIME ^5^ | Population abundance time series from the RivFishTIME database. Covering 2,386 abundance time series, derived from 40,834 abundance observations. These time series represent 197 unique sites and 191 species.  Temporal extent: 1975 - 2019  Latitude extent: -28.3 - 67.9  Longitude extent: -122.4 - 153.4 |
| F: UK riverine fishes ^6^ | Population abundance time series from the UK Environment Agency Fish population database, describing fish populations in rivers, lakes and transitional/coastal waters. Covering 361 abundance time series, derived from 3,016 abundance observations. These time series represent 181 unique sites and 16 species.  Temporal extent: 1984 - 2019  Latitude extent: 50.4 - 55.4  Longitude extent: -3.9 - 0.5 |
| G: Atlantic reef fishes ^10^ | Population abundance time series from the TimeFISH database, describing abundances of reef assemblages in the South-western Atlantic. Covering 86 abundance time series, derived from 262 abundance observations. These time series represent 12 unique sites and 52 species.  Temporal extent: 2008 - 2022  Latitude extent: -27.7 - -27.1  Longitude extent: -48.5 - -48.3 |
| H: German vegetation ^41^ | Population abundance time series from the ReSurveyGermany database, describing relative cover in vegetation plots. Covering 356 abundance time series, derived from 4,954 abundance observations. These time series represent 7 unique sites and 93 species.  Temporal extent: 1965 - 2018  Latitude extent: 48.3 - 53.6  Longitude extent: 7.4 - 13.9 |
| I: European biodiversity ^4^ | Population abundance time series from the Pilotto et al., (2020) study ‘’Meta-analysis of multidecadal biodiversity trends in Europe’ dataset - representing diverse taxa across the terrestrial, freshwater and marine realms. Covering 582 abundance time series, derived from 11,353 abundance observations. These time series represent 67 unique sites and 356 species.  Temporal extent: 1974 - 2018  Latitude extent: 40.1 - 67.8  Longitude extent: -8.9 - 29.6  Notes: This dataset was not openly available (unlike all others) and compiled, instead the dataset only provided the references to the primary sources. We extracted relative abundance/density estimates from across the 51 primary sources referenced within the database. We excluded a further 31 datasets contained within this database,  as the data lacked clear metadata, or data were not resolved to the species level, or data represented species presence/absences instead of abundances. |
| J: Large carnivores ^7^ | Population abundance time series from the CaPTrends database of large carnivore population trends and time series. Covering 279 abundance time series, derived from 2,670 abundance observations. These time series represent 165 unique sites and 26 species.  Temporal extent: 1880 - 2019  Latitude extent: -40.0 - 71.6  Longitude extent: -158.0 - 99.2 |

Convergence

Conventional Bayesian approaches assess model convergence by ensuring multiple independent Monte Carlo Markov chains arrive at a similar parameter space after thousands of iterations. As INLA approximates parameters, there are no chains to explore. Instead, to validate model convergence in INLA, we re-ran each model 10 times tracking changes in key parameters - the collective trend coefficient and associated uncertainty. The first time each model was run we used the randomly generated initial values but for each subsequent run, the initial values for each parameter were set as the parameter modes from the prior run. It was evident all models showed relative parameter stability by the 5th run (Figure S1).


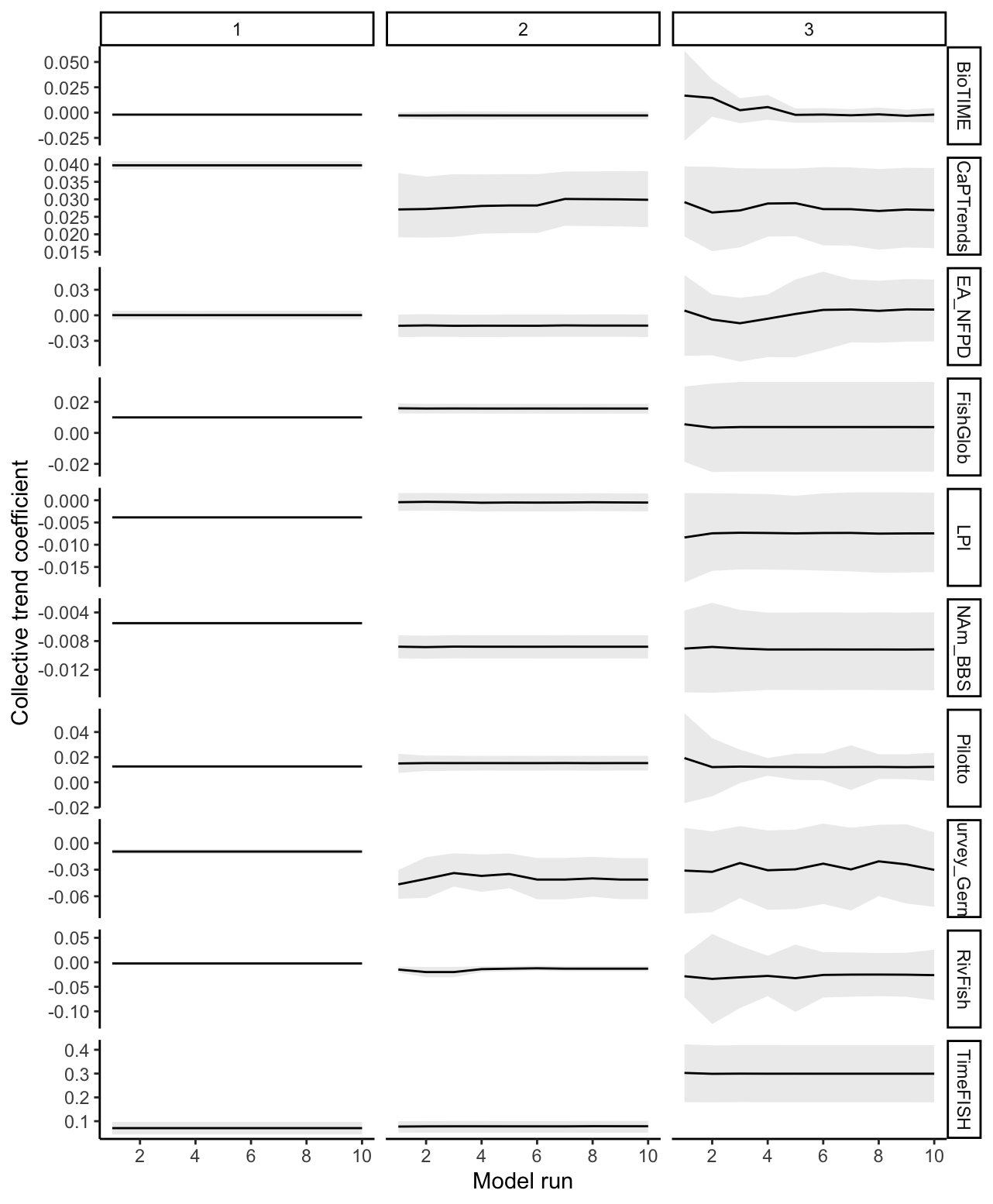


**Figure S1.** Change in median collective trend coefficients (plus 95% credible intervals) for each dataset under an increasing number of model runs. Parameters begin to stabilise by the 5th run. Model parameters are drawn from the following number of abundance observations: BioTIME (n = 243,993), Living Planet (n = 77,773), NA Breeding Birds (n = 164,137), FishGlob (n = 67,908), RivFishTIME (n = 40,834), UK riverine fishes (n = 3,016), Atlantic reef fishes (n = 262), German vegetation (n = 4,954), European biodiversity (n = 11,353), Large carnivores (n = 2,670).

Assumptions

We validated the assumptions of our model by ensuring residuals exhibited relative homoscedasticity and normality. To ensure homoscedasticity, we plotted the yearly median and quantiles (50% and 95%) of the residuals for each dataset and model, and observed relative stability of variance in the residuals through time - suggesting the homoscedasticity assumption passes. To test for normality of residuals, we plotted residual histograms for each dataset and model combination, finding normal distributions with slightly heavy tails in most datasets. These heavy tails have been highlighted in prior work, and are caused by the presence of extreme population dynamics (like population cycles; Figure S2-5), or extreme/erroneous population values (populations decline 99% in one year, and recover almost completely a year later; Figure S4). Extreme dynamics like this are likely present in all marco-scale biodiversity change models, and moving forward, future research should consider how best to handle these dynamics. Imposing a strict cut off - only keeping the 50% best fitting populations (i.e. removing the half of the populations with poor fit), and removing all residuals outside of two standard deviations from the mean residual within a population (i.e. the 5% worst fitting values within a population time series) - reduced the heavy-tails. But this introduces a trade-off that the spatial and taxonomic extents will also reduce. Notably, for our analysis, we expect and show that partial violation of the normality assumption has no substantial impact on our conclusions inference. Specifically, imposing the strict cut-off described above to reduce the heavy tails in the most affected dataset (Living Planet), under re-analysis we still see the same pervasive pattern where uncertainty around trends increase as you move from the random intercept (standard deviation of collective trend = 0.0002) to the random slope (standard deviation of collective trend = 0.005), and through to the correlated effect model (standard deviation of collective trend = 0.05). This consistency in findings is not surprising though, as linear regressions are generally considered robust to deviations from a normal error distribution under large sample sizes. In summary, we are confident that the presence of these extreme values, and the heavy tailed residuals they create, have no meaningful impact on the conclusions in this paper.

DarrelDarrell Hamptonl Hampton
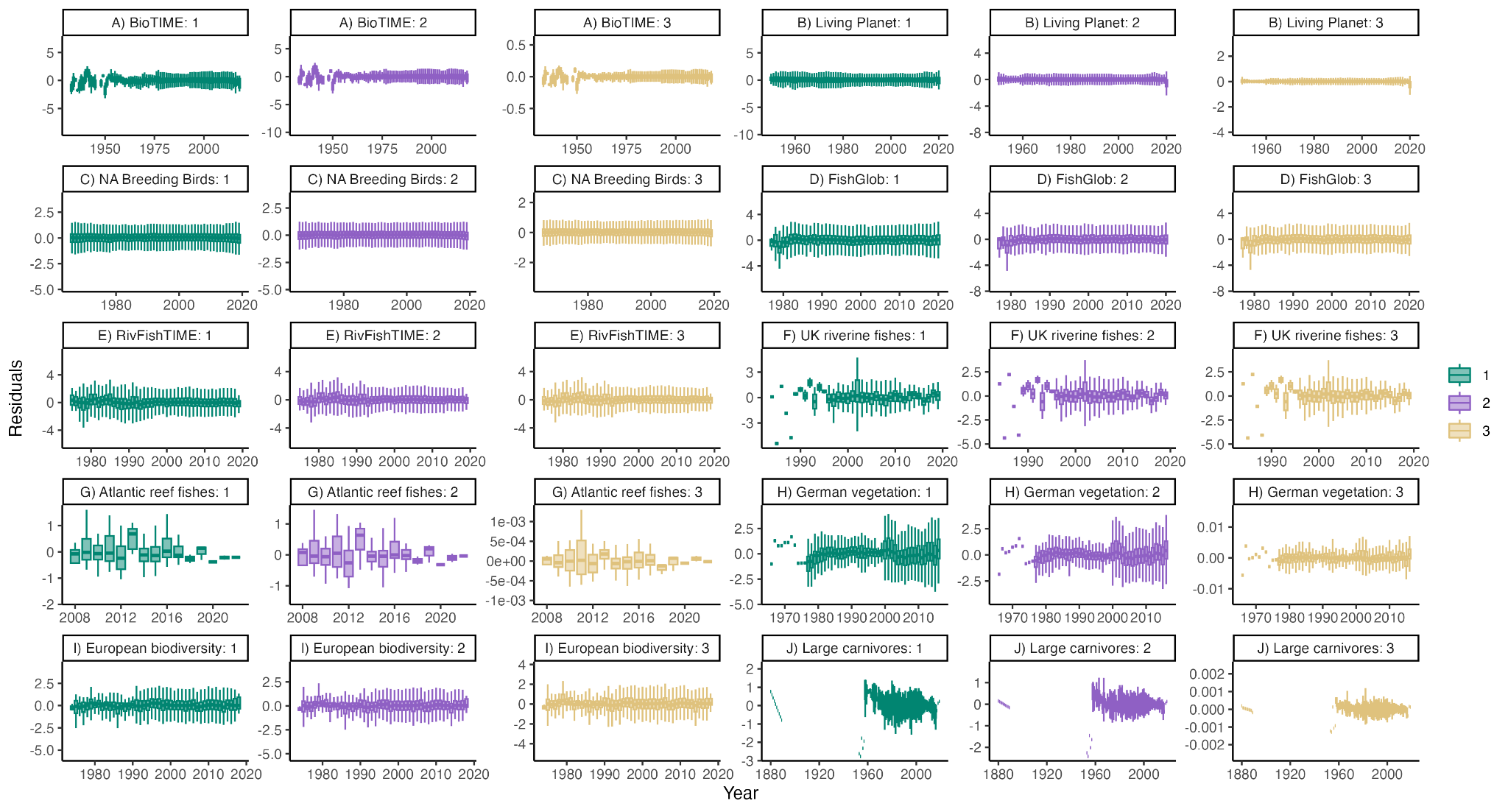


**Figure S2.** Boxplots depicting the median and quartiles (Box bounds at 50% quartiles and whiskers at 95% quartiles) of residuals through time for each dataset and model (1: Random intercept, 2: Random slope, 3: Correlated effect) combination. Boxplots are drawn from the following number of abundance observations: A) BioTIME (n = 243,993), B) Living Planet (n = 77,773), C) NA Breeding Birds (n = 164,137), D) FishGlob (n = 67,908), E) RivFishTIME (n = 40,834), F) UK riverine fishes (n = 3,016), G) Atlantic reef fishes (n = 262), H) German vegetation (n = 4,954), I) European biodiversity (n = 11,353), J) Large carnivores (n = 2,670).


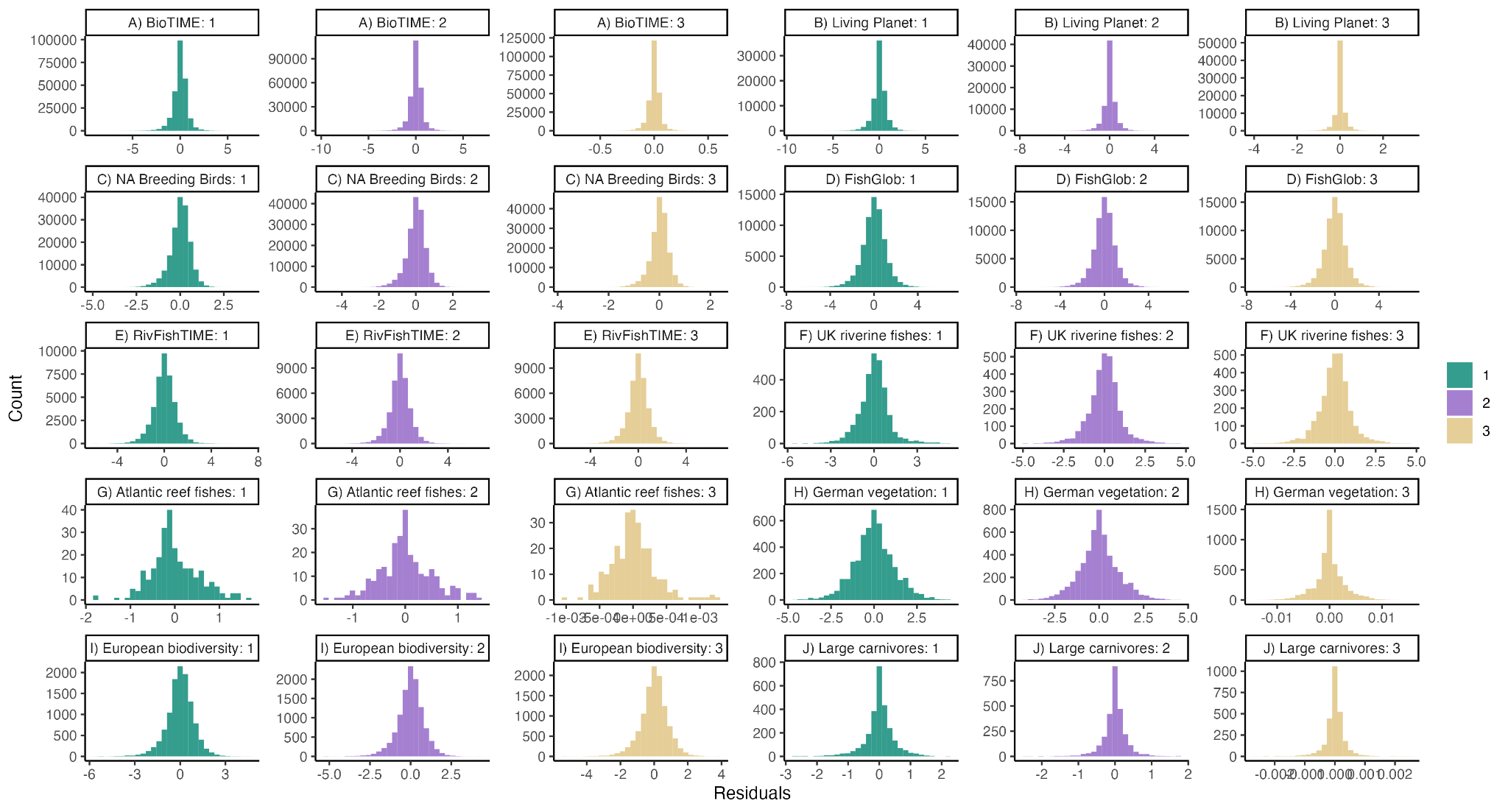


**Figure S3.** Distribution of residuals for each model (1: Random intercept, 2: Random slope, 3: Correlated effect) and dataset combination.


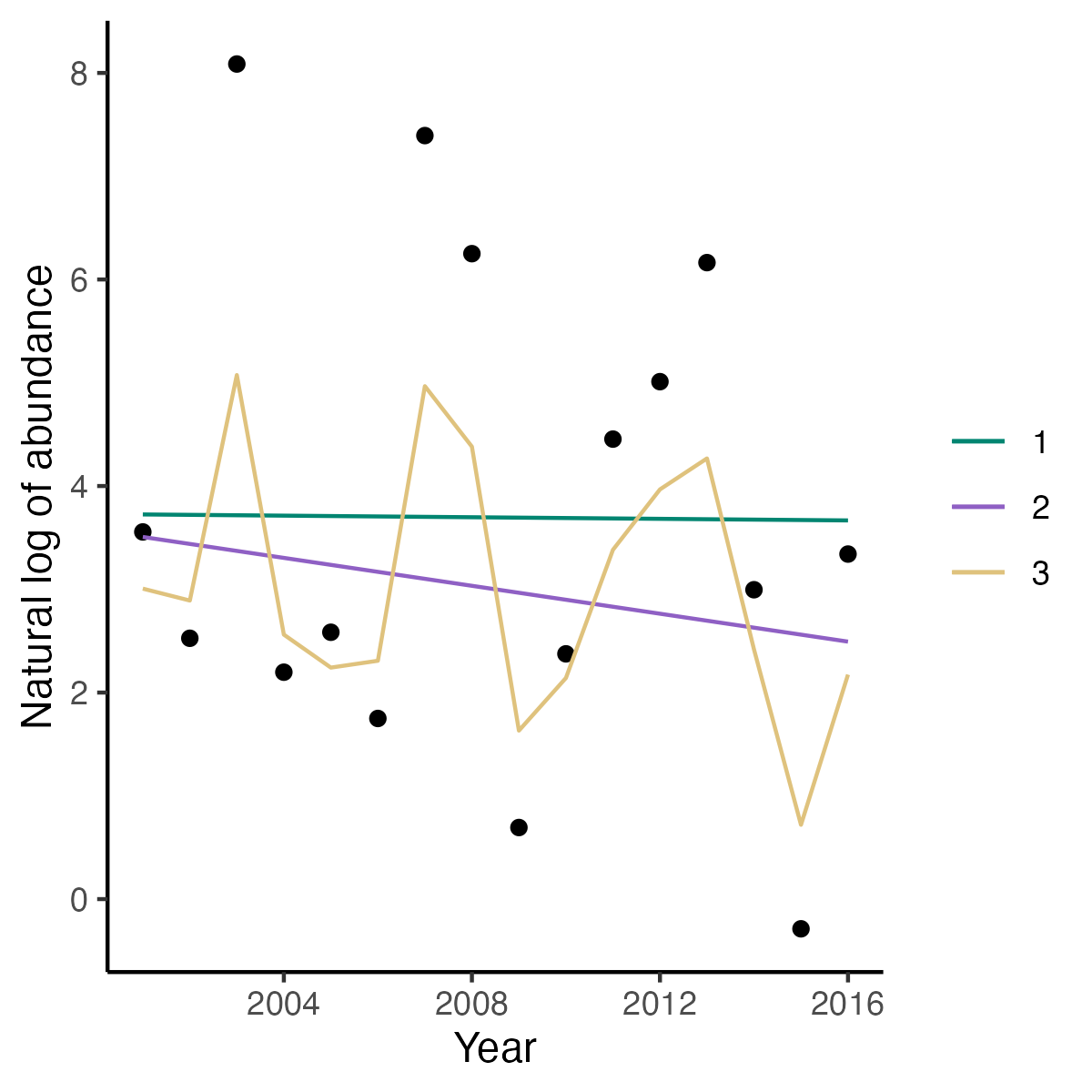

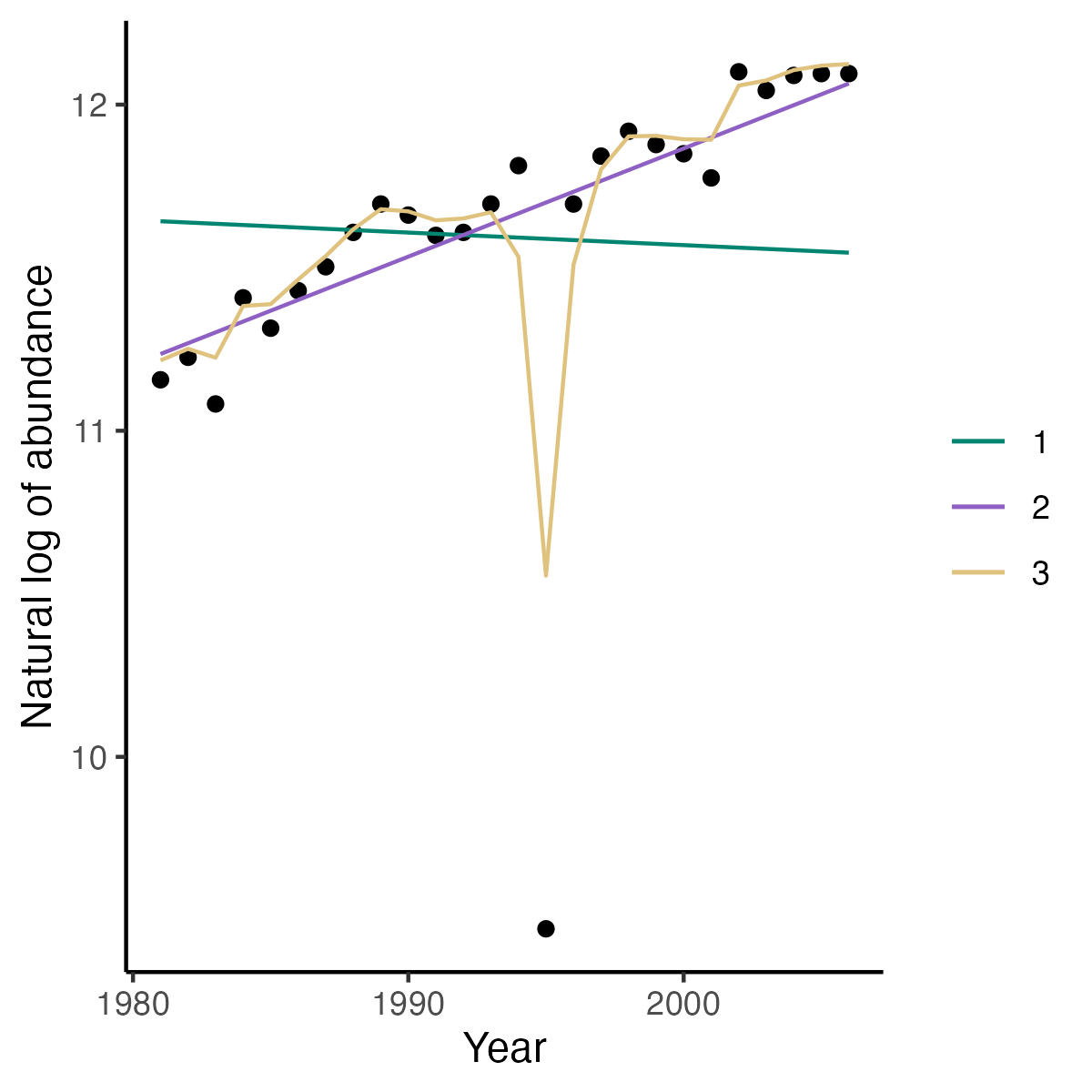


**Figure S4**. Left) Example of the models failing to fit to the observed abundances. In this case the population cycles, introducing a dynamic of extreme growth and loss, characteristics macro-scale models like ours are currently ill-equipped to capture. Right) An example where the model seemingly can represent the population dynamics, but with one extreme case where populations drop rapidly before sharply recovering. Both of these examples introduce heavy-tailed residuals.


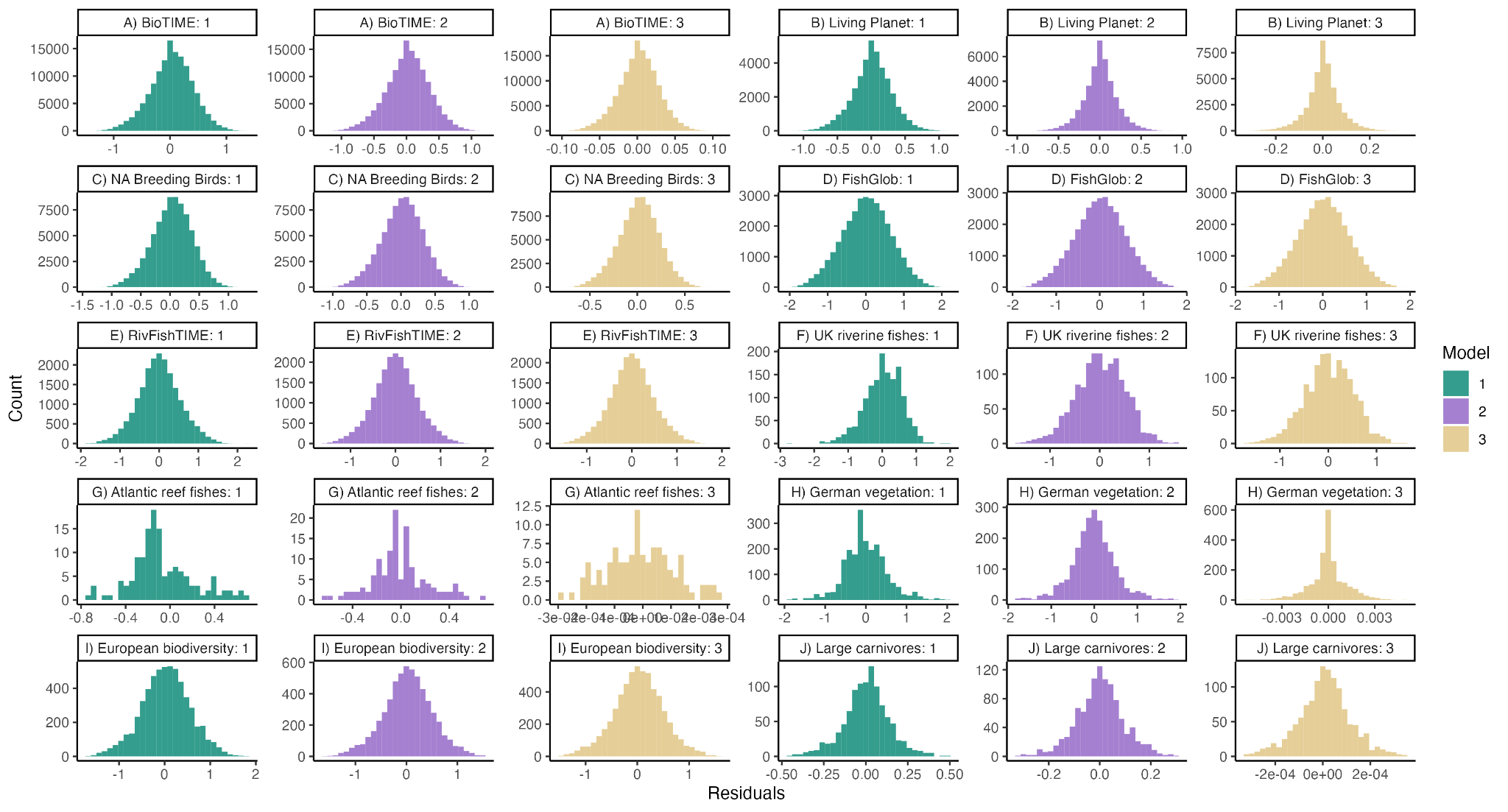


**Figure S5.** Distribution of residuals for each model (1: Random intercept, 2: Random slope, 3: Correlated effect) and dataset combination, after removing the 50% worst fitting populations (i.e. removing the half of the populations with poor fit), and removing all residuals outside of two standard deviations from the mean residual within a population (i.e. the 5% worst fitting values within a population time series)

Phylogeny

To account for correlative non-independence introduced by species' shared evolutionary past, we extracted a phylogeny for each dataset. For the phylogeny, we considered two options, each involving a trade-off: 1) We could use high quality phylogenies derived from genetic approaches, where branch lengths are known. However, this would introduce a trade-off, as many species lack phylogenetic information and so would have to be removed from the data. 2) The alternative was to adopt the framework developed by the Open Tree of Life. This framework merges phylogenies with taxonomies to produce approximate topologies (i.e. synthetic trees). By relaxing the quality constraints, the topologies have far greater taxonomic coverage (Table S3), but do lack branch lengths, potentially introducing error into phylogenetic models, as our ability to observe a phylogenetic signal is partially dependent on the quality of a phylogeny. If a phylogenetic signal is underestimated, which is possible under a poorer quality phylogeny, the variability around the collective abundance trend may also be misestimated. We conduct sensitivity analyses to understand how high quality phylogenies could change inference, and how restricting the data to purely high quality phylogeny species could alter inference on collective abundance trends. We consider three scenarios:

1. Using all available data and the OTL phylogeny
2. Using a restricted dataset, only containing species available in a smaller but higher quality TimeTree phylogeny (which contains branch lengths)
3. Using a restricted dataset, only containing species available in a smaller TimeTree phylogeny (which contains branch lengths), and trimming the OTL phylogeny to these species

Using higher quality phylogenies with branch lengths would reduce the taxonomic extent of the analyses, with more than 1000 species excluded across the 10 datasets (Table S3). Regarding inference (Figure S6), the collective trend coefficient is generally consistent amongst the phylogeny types (OTL: Open Tree of Life; TimeTree) and data restrictions (Full: All species available in the OTL; Restricted: Only species available in both OTL and TimeTree). The uncertainty around the collective trend is also largely consistent, especially under the competing phylogenies of B (OTL) and C (TimeTree). The greatest differences occur between A and B, which are driven by the loss of data. We hypothesise that the small differences in uncertainty around the collective trend between B and C are driven by the degree of variance captured by the spatial and phylogenetic random effects, where uncertainty increases around the collective trend when space and the phylogeny capture more of the variance in the data (Figure S7).

**Table S3.** Species count for each dataset under ‘Full: species available in the OTL’, and ‘Restricted: species in TimeTree”. Loss describes how many species would have to be excluded under the higher quality TimeTree phylogenies.

| **Dataset** | **Full** | **Restricted** | **Loss** |
| --- | --- | --- | --- |
| A: BioTIME | 1233 | 581 | 652 |
| B: Living Planet | 1264 | 751 | 513 |
| C: North American Breeding Birds | 361 | 290 | 71 |
| D: FishGlob | 152 | 133 | 19 |
| E: RivFishTIME | 191 | 158 | 33 |
| F: UK riverine fishes | 16 | 15 | 1 |
| G: Atlantic reef fishes | 52 | 50 | 2 |
| H: German vegetation | 93 | 85 | 8 |
| I: European biodiversity | 356 | 144 | 212 |
| J: Large carnivores | 26 | 25 | 1 |


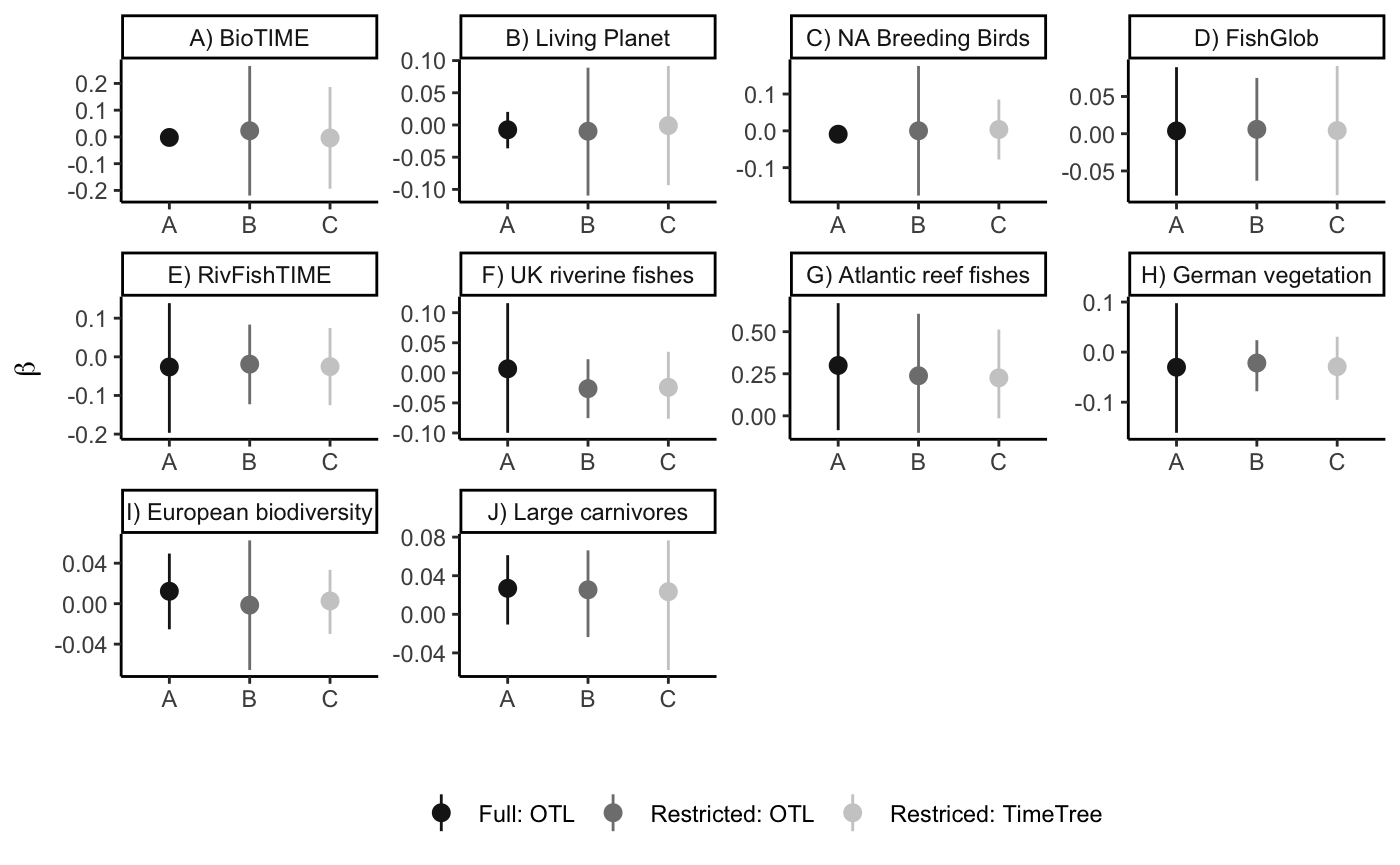


**Figure S6.** Median collective trend and associated 95% credible intervals across the 10 datasets under three conditions: A) Full OTL - using the OTL phylogeny and all available species; B) Restricted OTL - using the OTL phylogeny but only for species also available in the TimeTree phylogeny; C) Restricted TimeTree - using the TimeTree phylogeny and only species available in the TimeTree phylogeny. Model error and uncertainty is drawn from the following number of abundance observations: BioTIME (n = 243,993), Living Planet (n = 77,773), NA Breeding Birds (n = 164,137), FishGlob (n = 67,908), RivFishTIME (n = 40,834), UK riverine fishes (n = 3,016), Atlantic reef fishes (n = 262), German vegetation (n = 4,954), European biodiversity (n = 11,353), Large carnivores (n = 2,670)


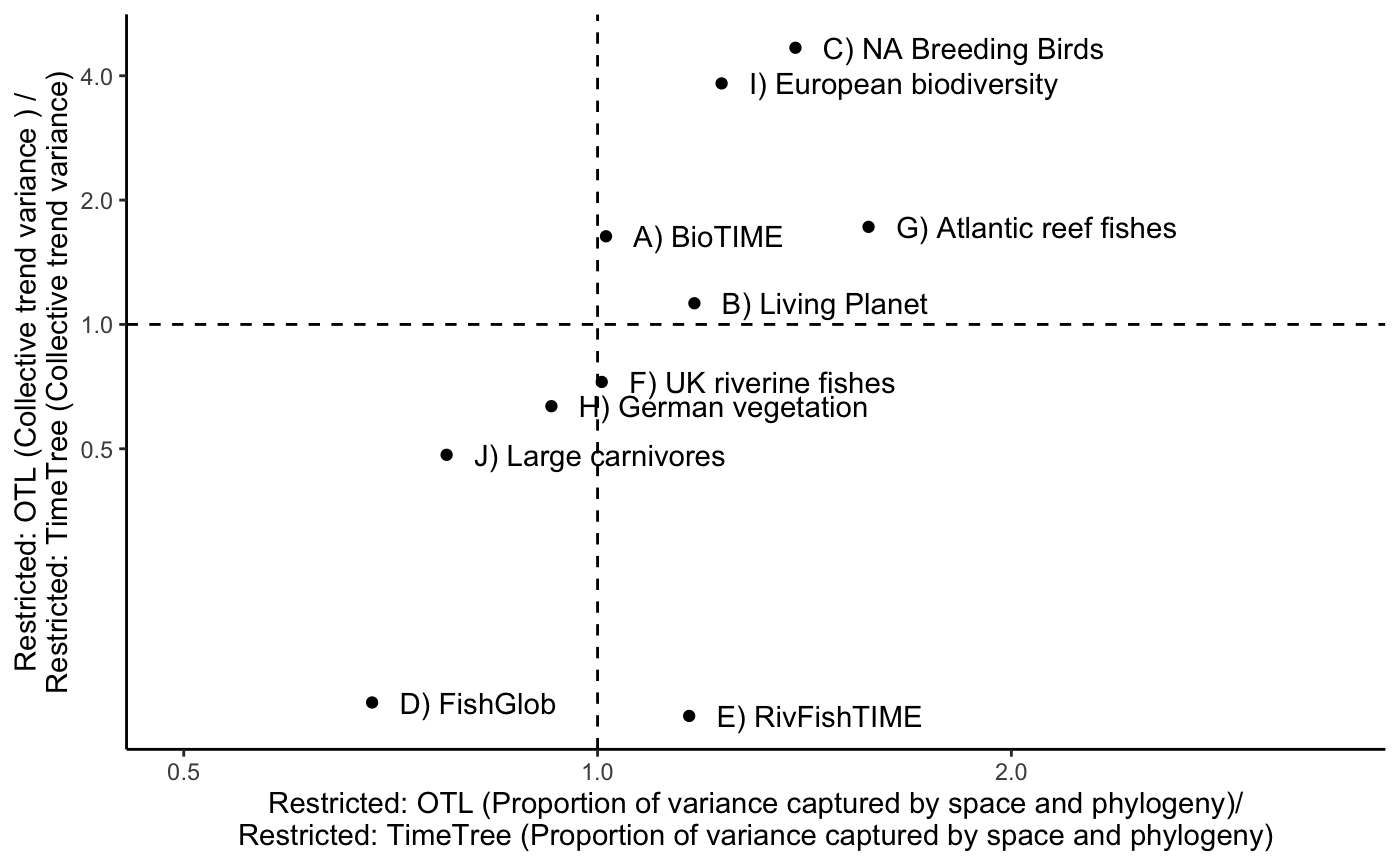


**Figure S7.** Collective trend variance under the OTL phylogeny divided by collective trend variance under the TimeTree phylogeny, plotted against the relative proportion of variance captured by the spatial and phylogenetic terms under the two phylogenies i.e. OTL variance captured divided by TimeTree variance captured.

Component importance

To explore how the addition of each correlative term changes inference, especially uncertainty around the collective trend, we iteratively integrate each collective term into the random slope model, producing the following combinations: Random slope + phylogeny, Random slope + time, Random slope + space, Random slope + space + time, Random slope + time + phylogeny, Random slope + space + phylogeny, Random slope + space + time + phylogeny. We compare the collective trend uncertainty in the collective trend under these models to the simple Random slope model. Our results show that accounting for correlative effects can substantially change uncertainty, particularly the introduction of spatial terms which lead to the greatest increase in collective trend uncertainty (Extended data Fig S2).

**Assorted figures and tables**

**Table S4.** Collective trend coefficients (Mean) under each model (1: Random intercept, 2: Random slope, 3: Correlated effect) and dataset, with associated standard deviation (SD), 50% credible intervals (CI) and 95% credible intervals.

| **Dataset** | **Model** | **Mean** | **SD** | **Lower 50% CI** | **Upper 50% CI** | **Lower 95% CI** | **Upper 95% CI** |
| --- | --- | --- | --- | --- | --- | --- | --- |
| **A) BioTIME** | 1 | -0.002 | 0.00021 | -0.0022 | -0.0019 | -0.0025 | -0.0016 |
|  | 2 | -0.0028 | 0.0061 | -0.0069 | 0.0011 | -0.015 | 0.0092 |
|  | 3 | -0.002 | 0.011 | -0.0097 | 0.0045 | -0.023 | 0.023 |
| **B) Living Planet** | 1 | -0.0039 | 0.00029 | -0.004 | -0.0037 | -0.0044 | -0.0033 |
|  | 2 | -0.00052 | 0.003 | -0.0025 | 0.0015 | -0.0065 | 0.0053 |
|  | 3 | -0.0074 | 0.014 | -0.016 | 0.0017 | -0.036 | 0.02 |
| **C) Breeding Birds** | 1 | -0.0055 | 0.00017 | -0.0056 | -0.0054 | -0.0058 | -0.0052 |
|  | 2 | -0.0088 | 0.0024 | -0.01 | -0.0072 | -0.014 | -0.004 |
|  | 3 | -0.0092 | 0.0086 | -0.015 | -0.004 | -0.026 | 0.0087 |
| **D) FishGlob** | 1 | 0.01 | 0.00046 | 0.0097 | 0.01 | 0.0091 | 0.011 |
|  | 2 | 0.016 | 0.0048 | 0.012 | 0.019 | 0.0065 | 0.025 |
|  | 3 | 0.0038 | 0.044 | -0.025 | 0.033 | -0.083 | 0.089 |
| **E) RivFishTIME** | 1 | -0.0024 | 0.00081 | -0.0029 | -0.0018 | -0.0039 | -0.00077 |
|  | 2 | -0.013 | 0.0082 | -0.018 | -0.0074 | -0.03 | 0.0027 |
|  | 3 | -0.026 | 0.084 | -0.077 | 0.026 | -0.2 | 0.14 |
| **F) UK Riverine Fishes** | 1 | 0.00017 | 0.0076 | -0.005 | 0.0053 | -0.015 | 0.015 |
|  | 2 | -0.012 | 0.021 | -0.026 | 0.00079 | -0.052 | 0.03 |
|  | 3 | 0.0067 | 0.055 | -0.031 | 0.042 | -0.1 | 0.12 |
| **G) Atlantic Reef Fishes** | 1 | 0.071 | 0.039 | 0.044 | 0.097 | -0.0063 | 0.15 |
|  | 2 | 0.079 | 0.04 | 0.052 | 0.1 | 0.0096 | 0.17 |
|  | 3 | 0.3 | 0.19 | 0.18 | 0.42 | -0.085 | 0.67 |
| **H) German Vegetation** | 1 | -0.0095 | 0.0038 | -0.012 | -0.007 | -0.017 | -0.0021 |
|  | 2 | -0.041 | 0.036 | -0.063 | -0.017 | -0.12 | 0.028 |
|  | 3 | -0.03 | 0.066 | -0.072 | 0.012 | -0.16 | 0.098 |
| **I) European Biodiversity** | 1 | 0.013 | 0.0012 | 0.012 | 0.013 | 0.01 | 0.015 |
|  | 2 | 0.015 | 0.0091 | 0.0094 | 0.021 | -0.0027 | 0.033 |
|  | 3 | 0.012 | 0.018 | 0.001 | 0.024 | -0.025 | 0.05 |
| **J) Large Carnivores** | 1 | 0.04 | 0.0018 | 0.039 | 0.041 | 0.036 | 0.043 |
|  | 2 | 0.03 | 0.012 | 0.022 | 0.038 | 0.0045 | 0.053 |
|  | 3 | 0.027 | 0.018 | 0.016 | 0.039 | -0.011 | 0.061 |
|  |  |  |  |  |  |  |  |

**Table S5.** Breakdown of variance attribution across the datasets, proportional to total variance i.e. the sum of variance captured by the residuals, fixed effect, population slope, temporal ar-1 term, species terms (species slope, genus slope, species slope under phylogenetic covariance) and site terms (site slope, region slope, site slope under spatial covariance). Rho describes the average temporal autocorrelation between abundance values. Spatial contribution describes the proportion of variance captured the site slope under spatial covariance relative to variance captured by all site terms (site slope, region slope, site slope under spatial covariance). Phylogenetic contribution describes the proportion of variance captured by the species slope under phylogenetic covariance relative to variance captured by all species terms (species slope, genus slope, species slope under phylogenetic covariance).

| **Database** | **Residual variance** | **Fixed variance** | **Temporal variance** | **Rho** | **Site variance** | **Spatial contribution** | **Taxonomic variance** | **Phylogenetic contribution** |
| --- | --- | --- | --- | --- | --- | --- | --- | --- |
| A: BioTIME | 0.18 | 0.28 | 0.44 | 0.17 | 0.05 | 0.09 | 0.04 | 0.17 |
| B: Living Planet | 0.29 | 0.24 | 0.40 | 0.40 | 0.04 | 0.30 | 0.03 | 0.34 |
| C: North American Breeding Birds | 0.44 | 0.11 | 0.38 | 0.41 | 0.04 | 0.17 | 0.03 | 0.35 |
| D: FishGlob | 0.45 | 0.14 | 0.34 | 0.45 | 0.04 | 0.87 | 0.02 | 0.30 |
| E: RivFishTIME | 0.26 | 0.23 | 0.39 | 0.17 | 0.08 | 0.85 | 0.03 | 0.21 |
| F: UK riverine fishes | 0.72 | 0.07 | 0.01 | 0.99 | 0.16 | 0.34 | 0.04 | 0.28 |
| G: Atlantic reef fishes | 0.006 | 0.21 | 0.39 | -0.65 | 0.17 | 0.04 | 0.22 | 0.94 |
| H: German vegetation | 0.04 | 0.47 | 0.41 | 0.41 | 0.04 | 0.33 | 0.04 | 0.90 |
| I: European biodiversity | 0.006 | 0.44 | 0.48 | 0.21 | 0.04 | 0.39 | 0.03 | 0.20 |
| J: Large carnivores | 0.001 | 0.32 | 0.49 | 0.40 | 0.14 | 0.07 | 0.04 | 0.36 |

**Table S6.** Comparison of each model's ability to recover missing abundance observations, Missingness imposed by removing the final abundance observation from half of the time-series in each dataset. These missing values are then predicted within each model and the percentage error calculated i.e. the absolute of the observed (the value we have removed) minus predicted abundance value, divided by the true value.

| **Dataset** | **Random intercept** | **Random slope** | **Correlated effect** |
| --- | --- | --- | --- |
| A: BioTIME | 15.9 | 13.0 | 12.7 |
| B: Living Planet | 11.2 | 7.09 | 6.24 |
| C: North American Breeding Birds | 17.7 | 15.0 | 14.6 |
| D: FishGlob | 10.8 | 9.63 | 9.63 |
| E: RivFishTIME | 24.1 | 20.3 | 20.0 |
| F: UK riverine fishes | 30.5 | 21.8 | 22.8 |
| G: Atlantic reef fishes | 54.8 | 38.1 | 22.9 |
| H: German vegetation | 49.7 | 32.7 | 27.3 |
| I: European biodiversity | 21.5 | 18.9 | 19.8 |
| J: Large carnivores | 8.12 | 6.39 | 5.61 |

**Table S7.** Ability of the random slope and correlated effect models to predict the population trends removed from the BioTIME dataset. We report the percentage error i.e. the absolute of the observed (the value of the trend we have removed) minus predicted trend value, divided by the true value. We report errors across the 50 leave-one-out iterations..

| **Dataset** | **Random slope full** | **Correlated effect full** |
| --- | --- | --- |
| A: BioTIME | 50.4 | 50.5 |
| B: Living Planet | 8.8 | 5.3 |
| C: North American Breeding Birds | 16.1 | 10.0 |
| D: FishGlob | 15.5 | 16.7 |
| E: RivFishTIME | 12.5 | 12.0 |
| F: UK riverine fishes | 92.0 | 44.5 |
| G: Atlantic reef fishes | 27.7 | 27.1 |
| H: German vegetation | 34.6 | 27.4 |
| I: European biodiversity | 15.8 | 11.5 |
| J: Large carnivores | 15.34 | 9.1 |

**References**

56. Loossens, T., Tuerlinckx, F. & Verdonck, S. A comparison of continuous and discrete time modeling of affective processes in terms of predictive accuracy. *Sci. Rep.* **11**, 6218 (2021).

57. Bakka, H. *et al.* Spatial modeling with R-INLA: A review. *WIREs Comput. Stat.* **10**, e1443 (2018).

58. Freckleton, R. P. The seven deadly sins of comparative analysis. *J. Evol. Biol.* **22**, 1367–1375 (2009).

59. Gelman, A. Prior Distribution for Variance Parameters in Hierarchical Models. *Bayesian Anal.* (2006).

60. Simpson, D., Rue, H., Riebler, A., Martins, T. G. & Sørbye, S. H. Penalising Model Component Complexity: A Principled, Practical Approach to Constructing Priors. *Stat. Sci.* **32**, 1–28 (2017).

61. Sørbye, S. H. & Rue, H. Penalised Complexity Priors for Stationary Autoregressive Processes. *J. Time Ser. Anal.* **38**, 923–935 (2017).

62. Wang, X., Yue, Y. & Faraway, J. *Bayesian Regression Modelling with INLA*. (CRC Press, 2018).

63. Evans, L. C. *et al.* Bioclimatic context of species’ populations determines community stability. *Glob. Ecol. Biogeogr.* **31**, 1542–1555 (2022).

64. Moraga, P. *Geospatial Health Data: Modeling and Visualization with R-INLA and Shiny*. (CRC Press).

65. Widdicombe, C. E., Eloire, D., Harbour, D., Harris, R. P. & Somerfield, P. J. Long-term phytoplankton community dynamics in the Western English Channel. *J. Plankton Res.* **32**, 643–655 (2010).

66. Holmes, R. T. & Sherry, T. W. Thirty-Year Bird Population Trends in an Unfragmented Temperate Deciduous Forest: Importance of Habitat Change. *The Auk* **118**, 589–609 (2001).

67. Williamson, M. The Land-Bird Community of Skokholm: Ordination and Turnover. *Oikos* **41**, 378–384 (1983).

68. Vickery, W. L. & Nudds, T. D. Detection of Density-Dependent Effects in Annual Duck Censuses. *Ecology* **65**, 96–104 (1984).

69. Linden, H. & Rajala, P. Fluctuations and long-term trends in the relative densities of tetraonid populations in Finland, 1964-77. *Finn. Game Res.* 13–14 (1981).

70. Pulliainen, E. A transect survey of small land carnivore and red fox populations on a subarctic fell in Finnish Forest Lapland over 13 winters. *Ann. Zool. Fenn.* **18**, 270–278 (1981).

71. Willig, M. R. & Bloch, C. P. El Verde Grid long-term invertebrate data: Luquillo Long Term Ecological Research Site Database: Data Set 107. (2016).

72. Friggens, M. Sevilleta LTER Small Mammal Population Data. (2008).

73. Lathrop, S. North Temperate Lakes LTER: Phytoplankton-Madison Lakes Area. (2005).

74. LTER, N. North Temperate Lakes LTER: Fish Abundance 1981-current. (2015).

75. LTER, N. T. L. North Temperate Lakes LTER: Zooplankton-Madison Lakes Area. (2005).

76. LTER, N. North Temperate Lakes LTER: Zooplankton-Trout Lake Area 1982-current. (2013).

77. Waide, R. Bird abundance-point counts, El Verde Field Station, Puerto Rico: Luquillo Long Term Ecological Research Site Database: Data Set 23. *Available Httpluq Lternet Edudataluqmetadata23 Accessed* (2012).

78. Ernest, S. M., Valone, T. J. & Brown, J. H. Long‐term monitoring and experimental manipulation of a Chihuahuan Desert ecosystem near Portal, Arizona, USA: Ecological Archives E090‐118. *Ecology* **90**, 1708–1708 (2009).

79. Moore, N. The development of dragonfly communities and the consequences of territorial behaviour: a 27 year study on small ponds at Woodwalton Fen, Cambridgeshire, United Kingdom. *Odonatologica* **20**, 203–231 (1991).

80. Zettler, M. Macrozoobenthos Baltic sea (1980–2005) as part of the IOW-Monitoring. *Inst. Für Ostseeforschung Warn. Ger.* 1980–2005 (2005).

81. Bakker, C., Herman, P. & Vink, M. A new trend in the development of the phytoplankton in the Oosterschelde (SW Netherlands) during and after the construction of a storm-surge barrier. *Oosterschelde Estuary Neth. Case-Study Chang. Ecosyst.* 79–100 (1994).

82. Reichert, M. MARMAP Chevron Trap Survey 1990-2009. *SCDNRNOAA MARMAP Program SCDNR MARMAP Aggreg. Data Surv. Mar. Resour. Monit. Assess. Predict. MARMAP Program Mar. Resour. Res. Inst. S. C. Dep. Nat. Resour.* (2009).

83. Reichert, M. MARMAP Florida Antillean Trap Survey 1990-2009. *SCDNR/NOAA* **30**,.

84. Reichert, M. MARMAP Blackfish Trap Survey 1990-2009. *SCDNRNOAA MARMAP Program SCDNR MARMAP Aggreg. Data Surv. Mar. Resour. Monit. Assess. Predict. MARMAP Program Mar. Resour. Res. Inst. S. C. Dep. Nat. Resour. USA Available Httpwww Usgs Govobis-Usa Accessed* (2013).

85. Kennedy, M. & Spry, J. Atlantic Zone Monitoring Program Maritimes Region plankton datasets. *Fish. Oceans Can.-BioChem Arch. OBIS Can. Bedford Inst. Oceanogr. Dartm. N. S. Can.* (2011).

86. Moore, J. & Howson, C. Survey of the rocky shores in the region of Sullom Voe, Shetland, A report to SOTEAG from Aquatic Survey & Monitoring Ltd. *Cosheston Pembs.*

87. Franklin, J. F. Long-term growth, mortality and regeneration of trees in permanent vegetation plots in the Pacific Northwest, 1910 to present. (2019).

88. McLarney, W., Meador, J. & Chamblee, J. Upper Little Tennessee River Biomonitoring Program Database. *Coweeta Long Term Ecol. Res. Program Available Httpscoweeta Uga Edudbpublicdatasetdetails Asp* (2012).

89. Gido, K. Fish population on selected watersheds at Konza Prairie-CFP01. *Konza Prairie LTER Program Available Httpwww Konza Ksu EduKNZpagesdataKnzdsdetail Aspx*.

90. Lathrop, R. Madison Wisonsin Lakes Zooplankton 1976-1994. (2013).

91. Day, F. P., Conn, C., Crawford, E. & Stevenson, M. Long-term effects of nitrogen fertilization on plant community structure on a coastal barrier island dune chronosequence. *J. Coast. Res.* **20**, 722–730 (2004).

92. Chen, H. *et al.* Long-term monitoring dataset of fish assemblages impinged at nuclear power plants in northern Taiwan. *Sci. Data* **2**, 1–6 (2015).

93. Shi, Z. *et al.* Evidence for long‐term shift in plant community composition under decadal experimental warming. *J. Ecol.* **103**, 1131–1140 (2015).

94. Thomsen, P. F. *et al.* Resource specialists lead local insect community turnover associated with temperature – analysis of an 18-year full-seasonal record of moths and beetles. *J. Anim. Ecol.* **85**, 251–261 (2016).

95. LTER, S. B. C. & Reed, D. C. SBC LTER: Reef: Kelp forest community dynamics: Invertebrate and algal density. (2018).

96. Reed, D. SBCLTER: Reef: Kelp Forest Community Dynamics: Abundance and SIze of Giant Kelp (Macrocystis Pyrifera), ongoing since 2000. (2010).

97. Reed, D. SBCLTER: Reef: Kelp Forest Community Dynamics: Fish Abundance. (2010).

98. Edgar, G. J. & Stuart-Smith, R. D. Systematic global assessment of reef fish communities by the Reef Life Survey program. *Sci. Data* **1**, 1–8 (2014).

99. Landis, D. & Gage, S. Insect Populations via Sticky Traps at KBS-LTER. (2014).

100. Wiley, R. Population estimates of Appalachian salamanders. (2005).

101. Merritt, J. Long Term Mammal Data from Powdermill Biological Station 1979-1999 (Reformatted to ecocomDP Design Pattern). (2019).

102. Kaufman, D. Seasonal summary of numbers of small mammals on 14 LTER traplines in prairie habitats at Konza Prairie. Konza Prairie Long-Term Ecological Research. (2016).

103. Knops, J. & Tilman, D. Successional Dynamics on a Resampled Chronosequence-Experiment 014. Cedar Creek Ecosystem Science Reserve. (2016).

104. LTER, J. B. Lizard pitfall trap data (LTER-II, LTER-III).

105. Lightfoot, D. & Schooley, R. SMES rodent trapping data, Small Mammal Exclosure Study. *Jorn. LTER Available Httpjornada Nmsu Edusitesjornada Nmsu EdufilesdatafilesJornadaStudy086smesrodenttrappingdata0 Csv Accessed* (2016).

106. Kelt, D., Meserve, P., Gutiérrez, J., Milstead, W. B. & Previtali, M. Long‐term monitoring of mammals in the face of biotic and abiotic influences at a semiarid site in north‐central Chile: Ecological Archives E094‐084. *Ecology* **94**, 977–977 (2013).

107. Davies, C. H. *et al.* Over 75 years of zooplankton data from Australia: Ecological Archives E095‐278. *Ecology* **95**, 3229–3229 (2014).

108. Grossman, G. D. Stream fish assemblage stability in a southern Appalachian stream at the Coweeta Hydrologic Laboratory from 1984 to 1995. (2013).

109. Svensson, S., Thorner, A. M. & Nyholm, N. E. I. Species trends, turnover and composition of a woodland bird community in southern Sweden during a period of fifty-seven years. *Ornis Svec.* **20**, 31–44 (2010).

110. Lightfoot, D. Small Mammal Exclosure Study (SMES) Vegetation Data from the Chihuahuan Desert Grassland and Shrubland at the Sevilleta National Wildlife Refuge, New Mexico (2006-2009). (2011).

111. Davies, C. H. *et al.* A database of marine phytoplankton abundance, biomass and species composition in Australian waters. *Sci. Data* **3**, 1–12 (2016).

112. Stapp, P. SGS-LTER long-term monitoring project: small mammals on trapping webs on the Central Plains Experimental Range, Nunn, Colorado, USA 1994-2006, ARS study number 118. (2013).

113. Dickson, J. G., Conner, R. N. & Williamson, J. H. Neotropical migratory bird communities in a developing pine plantation. in (1993).

114. Hall, G. A. A long-term bird population study in an Appalachian spruce forest. *Wilson Bull.* 228–240 (1984).

115. Enemar, A., Sjöstrand, B., Andersson, G. & Proschwitz, T. von. The 37-year dynamics of a subalpine passerine bird community, with special emphasis on the influence of environmental temperature and Epirrita autumnata cycles. *Ornis Svec.* **14**, 63–106 (2004).

116. Site, M. 1000 Project, Biodiversity Center, M. o. E. o. Japan,“Monitoring site 1000 Village survey-Medium and large mammal survey data (2006-2012)”. *SAT03zip Downloaded Httpwww Biodic Go Jpmoni1000findingsdataindex Html Accessed* **35**, 2016 (2014).

117. Benedetti-Cecchi, L. Calafuria Mid-shore Intertidal Dataset (1991-2014). *Dep. Biol. Univ. Pisa Accessed* 23–42 (2016).

118. Pollard, E. Monitoring butterfly numbers. in *Monitoring for conservation and ecology* 87–111 (Springer, 1991).

119. How, R. Long-term sampling of a herpetofaunal assemblage on an isolated urban bushland remnant, Bold Park, Perth. *J. R. Soc. West. Aust.* **81**, 143 (1998).

120. Krefting, L. W. & Ahlgren, C. E. Small mammals and vegetation changes after fire in a mixed conifer‐hardwood forest. *Ecology* **55**, 1391–1398 (1974).

121. Rossa-Feres, D. de C. Community ecology of anura amphibia at Northwest region of Sao Paulo state, Brazil: microhabitat, seasonality, diet and multidimensional niche. State University of São Paulo. (1997).

122. Svensson, S. Species composition and population fluctuations of alpine bird communities during 38 years in the Scandinavian mountain range. *Ornis Svec.* **16**, 183–210 (2006).

123. Pomati, F. *et al.* Challenges and prospects for interpreting long‐term phytoplankton diversity changes in Lake Zurich (Switzerland). *Freshw. Biol.* **60**, 1052–1059 (2015).

124. Barceló, C., Ciannelli, L., Olsen, E. M., Johannessen, T. & Knutsen, H. Eight decades of sampling reveal a contemporary novel fish assemblage in coastal nursery habitats. *Glob. Change Biol.* **22**, 1155–1167 (2016).

125. Zakharov, V. Biodiversity of bird population of terrestrial habitats in Southern Ural. *Miass Ilmenskiy Gos. Zapov. Im VI Lenina Ural. Otd. Ross. Akad. Nauk. Russ.* (1998).

126. Anderson, J., Vermeire, L. & Adler, P. B. Fourteen years of mapped, permanent quadrats in a northern mixed prairie, USA: Ecological Archives E092‐143. *Ecology* **92**, 1703–1703 (2011).

127. Douglass, J. G. *et al.* Seasonal and interannual change in a Chesapeake Bay eelgrass community: insights into biotic and abiotic control of community structure. *Limnol. Oceanogr.* **55**, 1499–1520 (2010).

128. Wagner, R., Marxsen, J., Zwick, P. & Cox, E. J. *Central European Stream Ecosystems: The Long Term Study of the Breitenbach*. (John Wiley & Sons, 2011).

129. Welti, E. A. R., Roeder, K. A., de Beurs, K. M., Joern, A. & Kaspari, M. Nutrient dilution and climate cycles underlie declines in a dominant insect herbivore. *Proc. Natl. Acad. Sci. U. S. A.* **117**, 7271–7275 (2020).
