# Supplementary material for: Revealing uncertainty in the status of biodiversity change
Source: Nature. 2024 Mar 27;628(8009):788–94. doi: 10.1038/s41586-024-07236-z (PMC11041640; doi:10.1038/s41586-024-07236-z)
Supplement: Supplementary file 2 — Reporting Summary [file 41586_2024_7236_MOESM2_ESM.pdf]

nature portfolio

Corresponding author(s): Thomas Frederick Johnson

Last updated by author(s): Feb 7, 2024

# Reporting Summary

Nature Portfolio wishes to improve the reproducibility of the work that we publish. This form provides structure for consistency and transparency in reporting. For further information on Nature Portfolio policies, see our Editorial Policies and the Editorial Policy Checklist.

## Statistics

For all statistical analyses, confirm that the following items are present in the figure legend, table legend, main text, or Methods section.

| n/a | Confirmed | |
|---|---|---|
| ☐ | ☒ | The exact sample size (*n*) for each experimental group/condition, given as a discrete number and unit of measurement |
| ☐ | ☒ | A statement on whether measurements were taken from distinct samples or whether the same sample was measured repeatedly |
| ☒ | ☐ | The statistical test(s) used AND whether they are one- or two-sided<br>*Only common tests should be described solely by name; describe more complex techniques in the Methods section.* |
| ☐ | ☒ | A description of all covariates tested |
| ☐ | ☒ | A description of any assumptions or corrections, such as tests of normality and adjustment for multiple comparisons |
| ☐ | ☒ | A full description of the statistical parameters including central tendency (e.g. means) or other basic estimates (e.g. regression coefficient) AND variation (e.g. standard deviation) or associated estimates of uncertainty (e.g. confidence intervals) |
| ☒ | ☐ | For null hypothesis testing, the test statistic (e.g. *F*, *t*, *r*) with confidence intervals, effect sizes, degrees of freedom and *P* value noted<br>*Give P values as exact values whenever suitable.* |
| ☐ | ☒ | For Bayesian analysis, information on the choice of priors and Markov chain Monte Carlo settings |
| ☐ | ☒ | For hierarchical and complex designs, identification of the appropriate level for tests and full reporting of outcomes |
| ☒ | ☐ | Estimates of effect sizes (e.g. Cohen's *d*, Pearson's *r*), indicating how they were calculated |

*Our web collection on statistics for biologists contains articles on many of the points above.*

## Software and code

Policy information about availability of computer code

Data collection

We have a fully reproducible and annotated data collection pipeline in the form of an RMarkdown document, named 'data_compile.Rmd' within our code repository (https://zenodo.org/records/10638241). Data was compiled from 10 sources (seed data availability below) and processed within R V4.0.5 using the following packages: tidyverse 2.0.0, here 1.0.1, janitor 2.2.0, countrycode 1.5.0 and arrow 12.0.1.

Arel-Bundock et al. (2018) countrycode: An R package to convert country names and country codes. Journal of Open Source Software https://doi.org/10.21105/joss.00848. Version 1.5.0

Firke S. (2023) janitor: Simple Tools for Examining and Cleaning Dirty Data. R package version 2.2.0, https://CRAN.R-project.org/package=janitor

Müller K. (2020) here: A Simpler Way to Find Your Files. R package version 1.0.1, https://CRAN.Rproject.org/package=here

R Core Team (2022) R: A language and environment for statistical computing. R Foundation for Statistical Computing, Vienna, Austria. https://www.R-project.org/. Version 4.0.5

Richardson, N. et al. (2023) arrow: Integration to 'Apache' 'Arrow'. R package version 11.0.0.3, https://CRAN.R-project.org/package=arrow

Wickham H. et al. (2019) Welcome to the tidyverse. Journal of Open Source Software https://doi.org/10.21105/joss.01686. Version 2.0.0

Data analysis

All of our code is openly available and reproducible from https://zenodo.org/records/10638241. Here, we provide a summary of our analysis, more information is available in the main text, methods, supplementary material and code.

The objective of our study was to consider how inference made in commonly applied biodiversity models could be impacted by failing to account for spatial, temporal and phylogenetic dependencies. Prior to designing our models, we first explored what models have been used in the literature to infer abundance change. In this work, we focussed on studies trying to characterise the average change in abundances over time, rather than studies attempting to assess how many species are declining or increasing, as this avoids discretizing a numeric value i.e. by assessing the average change, we avoid having to define what change is necessary to be classified as a 'decline'. To evaluate the diversity of approaches used to model abundance change over time in multi-species and/or multi-location datasets, we conducted a literature search within a selection of high profile ecology journals over the last 13 years. Our search identified 282 research papers, 28 of which described approaches to model abundance change across multi-species/location datasets. A further 16 methods were not detected within the literature search but were known priori to the authorship team, resulting in 44 different studies/methods. Models of abundance change varied in complexity, each containing their own assumptions, with no clear 'standard' approach for deriving the rate of change in abundance. However, across the 44 studies/methods we compiled (Table 1), five general approaches were present (Table 1):

Abundance average - The simplest models derive an average or total abundance across all species or sites in a given year, and then regress average abundance against time. This approach fails to recognise any of the hierarchical structure in the data.

Trend average - A slightly more complex model, which estimates abundance change per population by fitting a series of log-linear modes of abundance against year; averaging over the extracted slope coefficients. This approach fails to propagate uncertainty within average rates of change of each population, and ignores the implicit spatial and taxonomic structure within the data, inducing pseudoreplication.

Random intercept - Some studies partially address the aforementioned pseudoreplication (e.g. certain sites or species having multiple estimates) with mixed models, regressing log-linear abundance against year across all populations, whilst specifying that populations belong to a site and/or species. However, often this mixed model structure only extends to random intercepts , which only acknowledge that mean abundance can differ between sites, species and location, but assumes that the abundance trends will all remain the same. This is a particularly common approach amongst the indicators from population monitoring schemes which shape policy.

Random slope - In the scientific literature, it is common to use more complex models, with a similar structure to the Random intercept model, but now capturing the differences in abundance trends across populations, sites and species with random slope terms.

Decomposition - This is the rarest of the approaches and deviates from the linear mixed model approaches. Instead, the decomposition approach involves fitting generalised additive models (GAMs) through each time series to smooth abundance estimates and reduce noise. The smoothed time series is then decomposed into a timeseries of rates of change (or lambdas), which are then averaged across species and biomes to derive estimates of the average change in abundance for each year across all the time-series.

The most common approaches were the random intercept and random slope models, used 19 and 23 times, respectively. The abundance average, trend average and decomposition approaches were rare, used just 5, 2, and 3 times, respectively. Not all studies adopted just one approach, sometimes splitting their model into two steps e.g. using a random intercept model to estimate a given species trend across locations, which could then be aggregated across broader taxonomies with a random slope model. Further, all approaches regularly failed to recognise that abundance patterns are shaped by implicit temporal, spatial and phylogenetic signals (i.e. closely related species are likely to have more similar trends than distant species), with only 14 of the 44 approaches accounting for temporal autocorrelation. Phylogenetic and spatial covariance were comparably rarer - included in just 6 and 3 studies respectively. Four studies attempted to account for two sources of correlative non-independence within their models, by first deriving population trends whilst accounting for temporal autocorrelation of abundances within time series, and then using phylogenetic least squares to aggregate these trends. However, no study captured more than one of these covariances simultaneously (e.g. spatio-temporal models for instance). Further, no study attempted to account for all three sources of correlative non-independence at the same time.

Given the apparent rarity of the abundance average, trend average and decomposition approaches within the literature, we focus on understanding how the dominant approaches (i.e. the random intercept and random slope models) compare to our newly developed correlated effect model. Full model equations are available in 'Supplementary material - Models'.

Model 1. Random intercept

In model 1, we fit a linear mixed effect model between the natural logarithm of abundance and year, with five random intercepts: population (the unique time series), site (unique locations), region (broader spatial category to nest sites; measured as 10-degree grid cell the site occurs in), species (unique species), and genus (broader taxonomic category to nest species; measured as the parent node to the species tip). Within the model, we do not specify any nesting of the population within the site and species random intercepts as the hierarchical structure of the data is poorly defined e.g., whilst populations always occur within a species and site, some species are nested in sites, and some sites are nested in species, creating a crossed random effect design. Model 1 assumes all populations, sites, regions, species, and genera have the same trend in abundance.

Model 2. Random slope

In model 2, we develop a linear mixed effect model, where we regress the natural logarithm of abundance against year, including population, site, region, species, and genus all as random slopes. This builds on the random intercept model by allowing abundance-year slope coefficients to vary for each category in each random slope term (e.g., each species can have a different slope) - not simply differing intercepts as in model 1. Unlike model 1, we centre the year and abundances of each population time series at zero e.g. subtracting each year by the mean year in each population, and subtracting the log of each abundance by the mean log abundance value in each population. This centering fixes the y and x intercepts at zero for each slope, and is a convenient solution to acknowledge variance captured by the random intercepts without increasing the number of parameters. In all intents and purposes, the random slope model is equivalent to a model with random intercepts and slopes.

Model 3. Correlated effect

Model 3 is structurally similar to model 2, but accounts for correlative non-independence structures. For temporal non-independence, we model the population level time series with a discrete autoregressive-1 (ar-1) temporal process, which assumes neighbouring abundance observations within a time series will be more similar. To capture the spatial and phylogenetic correlative non-independence, we focus on non-independence across time series trends (instead of abundance observations), assuming trends in population abundances through time will be more similar in neighbouring sites and more closely related species. In model 1 and 2, we try to capture this non-independence with grouping categories (genus and region). However, in the correlated effect model, we more explicitly describe shared correlations between every species and site by specifying the covariance structure of our site and species random slopes. The site covariance matrix was derived by taking each site's coordinates and estimating the pairwise Haversine (spherical) distance between the sites e.g. how far is every site from every other site. This was then converted into a matrix, normalised between 0 and 1, with values close to 1 indicating neighbouring sites, whilst values approaching 0 indicate distant sites. The species covariance matrix was derived by converting the phylogeny into a variance-covariance matrix, where phylogenetic branch lengths describe the evolutionary distance between species.

All models were run in INLA using R V4.0.5. We describe our model priors in ‘Supplementary material - Priors’ and validate our model assumptions in ‘Supplementary material - Assumptions’. We also conduct additional sensitivity analyses exploring how phylogeny quality and how the addition of each correlative component (space, time or phylogeny) impacts inference - see ‘Supplementary material - Phylogeny’ and ‘Supplementary material - Component contribution’.

Outputs

Measuring non-independence
In our Correlated effect model we measure the presence of total non-independence as the proportion of variance captured by the combination of independent and correlative terms (i.e. random effects) for each component (e.g., temporal components), divided by the sum of the variance for all terms. Next, we assess if correlative terms are the larger contributor to this total non-independence, by dividing the proportion of variance captured by the correlated slopes, by the combination of the variance captured by the correlated and independent slopes. This was done separately for the spatial and phylogenetic terms. As the spatial and phylogenetic components each contain three terms (an independence species/location slope, an independent genera/region slope, and a correlated species/location slope), a proportional variance captured of 0.33 would indicate that the correlative slope captures an equal proportion of variance compared to the two independent slopes. A value greater than 0.33 indicates that correlative slopes account for more variation than independent random slopes. We measure temporal non-independence as the degree of correlation between neighbouring abundances (rho).

Differing inference between the models
Using the mean and 50% credible intervals of the global trend (overall abundance-time coefficients), we display abundance projections for each model in each dataset. These projections are based on an arbitrary baseline abundance of 100, set at the first year of available data in each dataset, and this abundance would change according to the overall coefficients and credible intervals. For instance, with a 1% annual rate of change, an abundance in year zero of 100, would become 101 in year 1, and 164 in year 50. The purpose of these projections is to showcase varying abundance trajectories under different model complexities.

Next, we note the number of the datasets where inference reverses (e.g., the global trend reverses direction from positive to negative, or remains consistent), and where uncertainty increases (the variance around the global trend is greater or smaller), comparing the random intercept and random slope models to the correlated effect model. To support these comparisons, we also report the fold change in the collective trend standard deviation of the correlated effect model, relative to the random intercept and random slope models.

Predictive performance
We assess the predictive performance of the different models by determining their ability to predict final observations in time series’, and their ability to predict population trends of a given species in a given location. To test the predictive accuracy for the final observation in the time series, we removed the final observation from half of the time-series in each dataset and predicted the missing values using each of the three models on the log scale. We report the percentage error (PE), a metric describing the median of the absolute percentage difference between predicted and observed values e.g. with a 5% error, an abundance on the log-scale of 1 would become 1.05. To test the accuracy of the population trend prediction, we conducted leave-one-out cross validation, removing one population time series (or trend) from each dataset, and estimating the missing trend using the random slope and correlated effect models. We repeated this process 50 times per dataset and compare the predicted trends to trends from a simplified correlated effect model, which contains a population level slope and accounts for temporal autocorrelation, but does not include the spatial and phylogenetic correlation terms or any of the hierarchical terms, which have no influence on the required population level inference. We measured trend predictive performance using the same approaches as above (PE). We estimate this error statistic across the full sample of 50 populations per dataset, and across the sub-sample of the rarest 20% of populations i.e. populations in the least well-studied species and location. In the random slope model, the population trend coefficients were derived by adding the species, location, genus, region, and overall coefficients together, meaning missing population values can still be informed by other hierarchical information. For the correlated effect model, the population trend is informed by the phylogenetic and spatial variance-covariance matrices, as well as all hierarchical information in the random slope model.

Phylogenetic and spatial distribution of abundance change
To plot abundance change across a phylogeny, we derived species level rates of change in abundance from the taxonomic (species and genera) and phylogenetic random effects. We incorporate uncertainty in species-level trend prediction by estimating the confidence interval threshold by which a species would be considered to have increased or decreased. For instance, a negative trend at an 80% confidence interval threshold would be considered stronger evidence of decline than a negative trend at at 20% interval threshold. We derive four asymptotic confidence interval thresholds (20%, 40%, 60%, 80%) using the uncertainty (standard deviation) from the phylogenetic random effect and a series of z-scores (0.25, 0.52, 0.84, 1.28).

To plot abundance change across space, we focus solely on one abundant and iconic species, the American Robin Turdus migratorius, as site-level trend variability is high at the community level i.e. community trends across space are rarely significant. To produce abundance change predictions for the American Robin across space, we expanded the BioTIME spatial Haversine distance matrix (describing distances between each time series) by supplementing it with a gridded extent covering North America. This new grid had a latitudinal range of 20 to 60 and 1 degree spacing (e.g. 15, 16, etc.), and longitudinal range of -130 to -60 with 1 degree spacing. This new matrix allows us to estimate expected

covariance (similarity) in abundance trends for any pair of 1 degree cells across North America. We then derived the average rate of change in abundance across all hierarchical and correlative random effects, and used population-level trend uncertainty to derive the selection of confidence interval thresholds described above.

For manuscripts utilizing custom algorithms or software that are central to the research but not yet described in published literature, software must be made available to editors and reviewers. We strongly encourage code deposition in a community repository (e.g. GitHub). See the Nature Portfolio guidelines for submitting code & software for further information.

## Data

Policy information about availability of data

All manuscripts must include a data availability statement. This statement should provide the following information, where applicable:

- Accession codes, unique identifiers, or web links for publicly available datasets
- A description of any restrictions on data availability
- For clinical datasets or third party data, please ensure that the statement adheres to our policy

All of the data used in the study are publicly available and accessible from the following links: RivFishTIME (https://doi.org/10.1111/geb.13210), North American Breeding Birds (https://doi.org/10.5066/P97WAZE5), BioTIME (https://doi.org/10.1111/geb.12729), Living Planet (https://www.livingplanetindex.org/data_portal), CaPTrends (https://doi.org/10.1111/geb.13587), ReSurvey Germany (https://doi.org/10.25829/idiv.3514-0qsq70), UK Fish Counts (https://environment.data.gov.uk/dataset/ce2618db-d507-4671-bafe-840b930d2297), FishGlob (https://doi.org/10.31219/osf.io/2bcjw), TimeFISH (https://doi.org/10.1002/ecy.3966), Pilotto (https://zenodo.org/records/10638241). See below for a summary of the data sources:

1) RivFishTIME

RivFishTime is a global database of freshwater fish time-series to study global change ecology in riverine systems, fully described in [Comte et al. 2020](https://doi.org/10.1111/geb.13210), with the data hosted by iDiv [here](https://idata.idiv.de/ddm/Data/ShowData/1873?version=12). Please see the full description and meta data of this database for more information.

The full zipped database can be downloaded directly and unzipped (before removing the zipped version). The two files required are the main survey dataset '1873_2_RivFishTIME_SurveyTable.csv' (this contains the record-level data, i.e. abundance of a given species at a given site in a given year), and the dataset describing each individual time series '1873_2_RivFishTIME_TimeseriesTable.csv' (this contains information on the specific location of each time series).

2) North American Breeding Bird Survey

The 2022 release of the North American Breeding Bird Survey dataset (1966-2021) is available from [Ziolkowski Jr. et al. (2022)](https://doi.org/10.5066/P97WAZE5). From the dataset description there: This dataset contains avian point count data for more than 700 North American bird taxa (species, races, and unidentified species groupings), collected annually during the breeding season along thousands of randomly established roadside survey routes in the United States and Canada. Routes are roughly 24.5 miles (39.2 km) long with counting locations placed at approximately half-mile (800-m) intervals, for a total of 50 stops. At each stop, a citizen scientist highly skilled in avian identification conducts a 3-minute point count, recording all birds seen within a quarter-mile (400-m) radius and all birds heard. Surveys begin 30 minutes before local sunrise and take approximately 5 hours to complete. Routes are sampled once per year, with the total number of routes sampled per year growing over time; just over 500 routes were sampled in 1966, while in recent decades approximately 3000 routes have been sampled annually. No data are provided for 2020. BBS field activities were cancelled in 2020 because of the coronavirus disease (COVID-19) global pandemic and observers were directed to not sample routes. Route location information includes country, state, and BCR, as well as geographic coordinates of route start point, and an indicator of run data quality. We require the 'States' and 'Routes' zipped datasets, and the species list (provided as a text file). The states data are provided as a zipped file for each state, but each of these is just a single csv file so can be read directly with `read_csv` To read them all into one big dataframe (specifying which columns to read, and stating their required data types). Processing involves adding location details and species names to this large dataset, creating a unique 'site' ID (from country, state, and route information), and mutating, renaming, or adding the other required variables:

3) BioTIME

BioTIME [(Dornelas et al. 2018)](https://doi.org/10.1111/geb.12729) is a comprehensive collection of assemblage time-series in which the abundances of the species that comprise ecological communities have been monitored over a number of years. BioTIME data span the globe and encompass land and seas; they also include freshwater systems. The current version of BioTIME contains over 12 million records, features almost 50 thousand species, covers over 600 thousand distinct geographic locations and is representative of over 20 biomes, occurring over 6 different climatic zones. This dataset requires registration prior to download. We downloaded the June 2021 version (the latest available) of the raw data in CSV format, together with the meta data and citations files, from the link provided following registration at https://biotime.st-andrews.ac.uk/download.php.
Processing the BioTIME data requires selecting relevant columns, and joining to the metadata in order to filter out studies that recorded only presence/absence, or only biomass (no abundance data). A 'site' variable is created by pasting the study ID, latitude, and longitude. No country information is provided.

4) Living Planet Index

The Living Planet Index (LPI; [LPI 2022](www.livingplanetindex.org/)) is a measure of the state of the world's biological diversity based on population trends of vertebrate species from terrestrial, freshwater and marine habitats. The LPI is based on trends of thousands of population time series collected from monitored sites around the world. Accessing the dataset requires registration prior to download from https://www.livingplanetindex.org/data_portal. We downloaded the latest available (2022) zipped version of the database into our raw data folder.

The subfolder 'LivingPlanetIndex_2022_PublicData' includes the LPI data agreement (data_agreement_2022.pdf) and metadata (LPD_metadata.pdf) as well as the public data as a csv file (LPD2022_public.csv). Read in this csv - NB 'NULL' is used to indicate missing abundance values. Country names are supplied, to convert

these to 3 character ISO codes we use the `countrycode` package [(Arel-Bundock et al. 2018)](https://doi.org/10.21105/joss.00848). There are two countries in the LPI data that do not match due to character encoding issues, plus 'International Waters', so we set up custom matches for these.

5) CaPTrends

CaPTrends ([Johnson et al. 2022](https://doi.org/10.1111/geb.13587)) is a database of 1,122 population trends from around the world, describing changes in abundance over time in large mammal species (n =50) from four families (Canidae, Felidae, Hyaenidae and Ursidae) in the order Carnivora. Trends represent 621 unique locations across the globe (latitude: −51.0 to 80.0; longitude: −166.0 to 166.0), from 1726 to 2017. The dataset itself is hosted on Zenodo here: https://zenodo.org/record/6949487. First, download the zipped dataset and unzip. Examining the meta data and descriptions, the two files we require are 'captrends.csv' (details of each individual study) and 'abundance.csv' (the actual abundance values). Some studies cover multiple countries - we assign the code to the first country mentioned. A 'site' variable is created by pasting `data_table_id`, `citation_key`, and `locality_name`. Latitude and longitude values are not available so these are set to NA. The abundance unit is set to the value in `population_metric` if available, otherwise to the `field_method` value.

6) ReSurvey Germany: vegetation-plot resurvey data from Germany

ReSurvey Germany: vegetation-plot resurvey data from Germany ([Jandt et al. 2022](https://doi.org/10.25829/idiv.3514-0qsq70)) is a compilation of harmonised vegetation-plot resurvey data from Germany covering almost 100 years. The data allow calculating temporal biodiversity change at the community scale. They also enable tracking changes in the incidence and distribution of individual species across Germany. Cover records are available for 1,794 vascular plant species in 7,738 (semi-)permanent vegetation plots from Germany, resurveyed from 2 to 54 times, in total resulting in 23,641 vegetation records and 458,311 species cover records, comprising the years from 1927 to 2020 and 97 EUNIS habitat types. The data is available to download from: https://doi.org/10.25829/idiv.3514-0qsq70. The main files required are the 'header' data (project / site level data) in 'Header_ReSurveyGermany.csv', and the main dataset of species-level abundances in 'ReSurveyGermany.csv'.

7) Environment Agency NFPD Fish Counts

The Environment Agency undertakes fisheries monitoring work on rivers, lakes and transitional and coastal waters (TraC) throughout England. The freshwater fish survey dataset (or _National Fish Populations Database_, NFPD, [Environment Agency 2020](https://environment.data.gov.uk/dataset/ce2618db-d507-4671-bafe-840b930d2297)) contains site and survey information, as well as the numbers and species of fish caught, for all the freshwater fish surveys carried out across England (with a small number from Wales and Scotland) from 1975 onwards. We accessed the Freshwater Fish Count dataset from https://environment.data.gov.uk/ecology/explorer/downloads/. Meta-data is available in a pdf document [here](https://environment.data.gov.uk/portalstg/sharing/rest/content/items/1150f6994d294d78b422b97848c3a286/data). The geographic coordinates in this database are provided as eastings and northings in the UK national projection ([EPSG 27700](http://www.opengis.net/def/crs/EPSG/0/27700)). In addition, there are some location errors placing surveys offshore or with incomplete coordinates (e.g. easting and northing both = 1). To address these, we use the `sf` library ([Pebesma 2018](https://doi.org/10.32614/RJ-2018-009)) to convert the database into spatial format, reproject to WGS84 latitude and longitude coordinates, and we combine it with a UK coastline map to exclude surveys that occur at sea. First, load `sf` and make the NFPD dataset spatial. We obtained the digital vector boundaries for Countries in the United Kingdom as at December 2022 at full resolution, clipped to the coastline (Mean High Water mark), from the Office for National Statistics, available https://geoportal.statistics.gov.uk/datasets/ons::countries-december-2022-uk-bfc/. We downloaded the shapefile, 'Countries_December_2022_UK_BFC_3731595901038458592.zip', directly downloaded and unzipped.

Now we can process this dataset. For the 'site' variable, there are two levels of site identification - the local survey site (identified by `site_id`, typically on the order of 100m river length) and a larger scale 'parent' site (`site_parent_id`), which may constitute several surveys within the same local area (typically all within a few km of each other). We retain both of these in a composite 'site' variable, meaning that the data are at the level of the site but that aggregation to parent site remains possible if required. Abundance is measured as the total number of fish sampled. This is done over one or more survey runs at each site in each time period. For around half the surveys, estimates of total population density are available by using the Carle & Strub equation ([Carle & Strub 1978](https://doi.org/10.2307/2530381)) over a three run catch depletion survey. However, this method can only be used on multiple run surveys, which would result in discarding over half available surveys, and so we take as our abundance measure the counts from the first run of the multiple run surveys, or the only run from single run surveys (as is done for Water Framework Directive classification; Philip Rudd, Fisheries Technical Specialist, Environment Agency, pers. comm. April 2023). Counts are then divided by the survey area (ength of river fished multiplied by the average width) to give abundance as individuals per 100 m^2^. For some surveys, exact counts are not given - these are excluded. Zero catches are recorded in a separate variable; these are converted to 0s in our abundance variable.

8) FishGlob Global Bottom Trawl Survey Database

FishGlob_data is a global database of bottom trawl survey data for marine fish, described in [Maureaud et al. (2023)](https://doi.org/10.31219/osf.io/2bcjw). The database contains a cleaned collation of 26 publicly available bottom-trawl surveys conducted in national waters of 18 countries that are standardised and pre-processed, covering a total of 2,162 sampled fish taxa and 232,800 hauls collected from 1963 to 2020. The database is available from Zenodo at https://zenodo.org/record/7527447#.ZDhrFuzMIqt. The full clean standardised dataset is found in the 'ouputs' subfolder as a .RData binary file, 'FishGlob_std_public_clean.RData'. This contains site level estimates of abundance per km2 from multiple bottom trawl surveys across multiple species. This data includes two objects, the main data as `data`, and meta-data in `readme`. Country is included in the dataset, but as country name. To convert to country code, create a dataframe of distinct countries, and use the `countrycode` ([Arel-Bundock et al. 2018](https://doi.org/10.21105/joss.00848)) package to obtain relevant ISO3 codes (all surveys listed as 'multi-countries' are in Europe, so we use EUROPE as a code for these).

Now process the data. As these abundances are derived through trawling, there is no discrete spatial repetition in sampling i.e. the exact same site is not sampled every year. Instead, the trawlers collecting data can deviate slightly from the exact site, but do often stay within the same general region/area. To handle these discrepancies in sampling location, we upscale the sampling locations (latitude and longitude) to a 1-degree resolution and take the average abundance estimates from the same sampling scheme,for each species in each year within this 1-degree gird cell. Simply put, we upscale the spatial resolution to allow temporal comparisons in trawling data. Note: A 1-degree resolution was decided on as it allowed us to develop these temporal comparisons whilst maintaining spatial structure

9) TimeFISH

The TimeFISH database ([Quimbayo et al. 2022](https://doi.org/10.1002/ecy.3966)) provides the first public time-series dataset on reef fish assemblages in the southwestern Atlantic (SWA), comprising 15 years of data (2007–2022) based on standardized Underwater Visual Censuses (UVCs) in nine locations along the southern Brazilian coast (25–29°S). All fish individuals in the water column (up to 2 m above the substratum) and at the bottom were targeted. In total, 202,965 individuals belonging to 163 reef fish species and 53 families were recorded across 1857 UVCs. Data are available to use with no restrictions, and can be downloaded from Zenodo: https://zenodo.org/record/7317084#.ZFJRK-zMIqs

Process the data. First, aggregate census data to species per transect (as we do not need individual size class abundances). Then fomat species names, and join to location data. Note - two transect IDs appear twice in the location data. As we are not interested in dates beyond years, we can disregard this in joining below by setting `multiple = "first"`. Then add dataset_id, create new site variable, add country code and unit, rename variables as needed and select the final set:

10) Pilotto

In 2020, Pilotto published a study on biodiversity trends in Europe, largely relaying on Europe's network of 'long-term ecological research sites'. Unlike the other datasets compiled in this Rmarkdown, Pilotto does not provide the full dataset, instead only sharing links to the raw data. To make use of the Pilotto data, we compiled this raw data into a series of .csv files (https://zenodo.org/records/10638241). Specifically, this involved working through the links available in Pilotto's data, downloading the raw data from these links into the directory 'data/raw_data/Pilotto/unique_id'. With each downloaded dataset, we then extracted information into three .csv files: compile_ts.csv - this file contains the abundance time-series, reported taxa, year, and name of the site; compile_sp.csv - this file contains the coordinates linked to sites contained in compile_ts.csv; and compile_tx.csv - this file contains species names, and is neccasary as reported taxa in 'compile_ts.csv' sometimes are described with codes instead of their actual name e.g. 'species_xyz123'. 'compile_tx.csv' can be used to convert these codes into actual species names. In some cases, we decided the information used by Pilotto was not suitable for our study (e.g. sometimes data contained presence/absence instead of abundance values). We have carefully annotated the data we extracted from Pilotto within the file 'Pilotto/master.csv'.

## Human research participants

Policy information about studies involving human research participants and Sex and Gender in Research.

| | |
|---|---|
| Reporting on sex and gender | NA |
| Population characteristics | NA |
| Recruitment | NA |
| Ethics oversight | NA |

Note that full information on the approval of the study protocol must also be provided in the manuscript.

# Field-specific reporting

Please select the one below that is the best fit for your research. If you are not sure, read the appropriate sections before making your selection.

☐ Life sciences ☐ Behavioural & social sciences ☒ Ecological, evolutionary & environmental sciences

For a reference copy of the document with all sections, see nature.com/documents/nr-reporting-summary-flat.pdf

# Ecological, evolutionary & environmental sciences study design

All studies must disclose on these points even when the disclosure is negative.

**Study description**

We assess how temporal trends in abundance change after accounting for spatial, temporal and phylogenetic dependencies. We test this across 10 high-profile datasets, reporting the change in abundance ~ year coefficient, and credible intervals. We also explore predictive accuracy after incorporating spatial, temporal and phylogenetic dependencies

**Research sample**

We use 10 high-profile abundance datasets, representing more than 30,000 populations, ~2,900 species and ~5,850 unique locations.

For each dataset, we extracted the population abundance estimates, the accompanying time-stamps, the species scientific names, the name of the site (location) where the population was sampled, and any site coordinates. For datasets to be included they had to be open access, and contain multiple abundance time series for a selection of species and locations. Whilst these datasets are vital within biodiversity science, many of the datasets are prone to biases e.g. lacking tropical representation, and contain few plant and invertebrate species. The datasets have been compiled from a variety of methods, realms and systems, covering a vast array of spatial, taxonomic and temporal scales. Further, there is likely some overlap in data between datasets - i.e. population time-series may occur in more than one dataset. We take no action to correct or acknowledge these biases and features, as our analysis is designed to show how model choice can have a substantial influence on inference in a variety of datasets, rather than to derive new trend estimates for each dataset or derive a consensus trend across datasets.

**Sampling strategy**

We selected datasets with open-use policies to enable the work to be reproducible. We conducted no power analyses to determine sample sizes as our work included all available data.

**Data collection**

See comprehensive instructions on data collection above.

**Timing and spatial scale**

The datasets vary in temporal, spatial and taxonomic scale. Most records tend to occur within later decades, the global north, and vertebrate populations. Please see a summary for each dataset below

Population abundance time series from the BioTIME dataset - representing all core taxa and realms. Covering 12,065 abundance time

series, derived from 243,993 abundance observations. These time series represent 438 unique sites and 1,233 species.
Temporal extent: 1933-2018
Latitude extent: -77.6 - 67.8
Longitude extent: -179.8 - 179.2

Global population abundance time series from the Living Planet database. Covering 5,255 abundance time series, derived from 166,827 abundance observations. These time series represent 1,159 unique sites and 1,264 species.
Temporal extent: 1950 - 2020
Latitude extent: -77.8 - 78.9
Longitude extent: -180 - 180

Population abundance time series from the North American breeding bird survey. Covering 8,718 abundance time series, derived from 164,317 abundance observations. These time series represent 584 unique sites and 361 species.
Temporal extent: 1966 - 2019
Latitude extent: 25.9 - 67.0
Longitude extent: -165.3 - -55.4

Population abundance time series from the FishGlob database, describing abundances from the bottom-trawl survey for marine fishes. Covering 2,286 abundance time series, derived from 67,908 abundance observations. These time series represent 229 unique sites and 152 species.
Temporal extent: 1977 - 2020
Latitude extent: 26 - 62
Longitude extent: -178 - 21

Population abundance time series from the RivFishTIME database. Covering 2,386 abundance time series, derived from 40,834 abundance observations. These time series represent 197 unique sites and 191 species.
Temporal extent: 1975 - 2019
Latitude extent: -28.3 - 67.9
Longitude extent: -122.4 - 153.4

Population abundance time series from the UK Environment Agency Fish population database, describing fish populations in rivers, lakes and transitional/coastal waters. Covering 361 abundance time series, derived from 3,016 abundance observations. These time series represent 181 unique sites and 16 species.
Temporal extent: 1984 - 2019
Latitude extent: 50.4 - 55.4
Longitude extent: -3.9 - 0.5

Population abundance time series from the TimeFISH database, describing abundances of reef assemblages in the South-western Atlantic. Covering 86 abundance time series, derived from 262 abundance observations. These time series represent 12 unique sites and 52 species.
Temporal extent: 2008 - 2022
Latitude extent: -27.7 - -27.1
Longitude extent: -48.5 - -48.3

Population abundance time series from the ReSurveyGermany database, describing relative cover in vegetation plots. Covering 356 abundance time series, derived from 4,954 abundance observations. These time series represent 7 unique sites and 93 species.
Temporal extent: 1965 - 2018
Latitude extent: 48.3 - 53.6
Longitude extent: 7.4 - 13.9

Population abundance time series from the Pilotto et al., (2020) study ''Meta-analysis of multidecadal biodiversity trends in Europe' dataset - representing diverse taxa across the terrestrial, freshwater and marine realms. Covering 2,386 abundance time series, derived from 40,834 abundance observations. These time series represent 197 unique sites and 191 species. Note: The compiled form of this dataset was not openly available (unlike all others), instead the dataset only provided the references to the primary sources. We extracted relative abundance/density estimates from across the 51 primary sources referenced within the database. We excluded a further 31 datasets contained within this database, as the data lacked clear metadata, or data were not resolved to the species level, or data represented species presence/absences instead of abundances.
Temporal extent: 1975 - 2019
Latitude extent: 40.1 - 67.8
Longitude extent: -8.9 - 29.6

Population abundance time series from the CaPTrends database of large carnivore population trends and time series. Covering 279 abundance time series, derived from 2,670 abundance observations. These time series represent 165 unique sites and 26 species.
Temporal extent: 1880 - 2019
Latitude extent: -40.0 - 71.6
Longitude extent: -158.0 - 99.2

Data exclusions

For each dataset we extract synthetic trees from the open tree of life 45,46 and estimate missing branch lengths using Grafen's approach 47 from the compute.brlen function in the R package ape 48. The Open Tree of Life identified a phylogeny for 80% of species (N = 23,871); all other species were removed from the analysis. For studies with the overall aim of assessing biodiversity change, removing species could be problematic, as the collective trend would not be representative of all species. However, in our case, where the aim is to assess how collective trend inference changes under a variety of modelling approaches, trimming the data to species with an accompanying phylogeny has no impact on our conclusions.

After removing species not present in the Open Tree of Life topology, we further trimmed the data to only include higher-quality time series, removing the following: time series that contained zeros (which we considered extreme cases of extinctions or recolonisations) and time series with missing abundance values for a given year throughout the sampling duration (i.e., we required consecutive abundance estimates.) In all datasets except the two smallest - G) Atlantic reef fishes & J) Large carnivores) - we further trimmed the datasets to only keep time series which had greater than or equal to the median number of abundance observations i.e., including the longest 50% of time series in each dataset. In some cases, this cut-off was not sufficient as the median number of observations in the time series equaled two. With only two abundance observations, trends are highly exposed to error purely driven by random fluctuations in abundance 10. To partially address this issue, we imposed a further cut-off on these datasets, ensuring each time-series had at least 5 observations. These datasets are characterised in Table S1. With our trimmed dataset, we derived a mean abundance in each year (in cases where there were more than one observation per year) for each time series.

Reproducibility

All code and code are openly avalable and reproducible

Randomization

Data was not randomized. Our study showcases the need to conduct statistical controls for non-independence, which has been been neglected in previous work

Blinding

NA

Did the study involve field work? ☐ Yes ☒ No

# Reporting for specific materials, systems and methods

We require information from authors about some types of materials, experimental systems and methods used in many studies. Here, indicate whether each material, system or method listed is relevant to your study. If you are not sure if a list item applies to your research, read the appropriate section before selecting a response.

## Materials & experimental systems

| n/a | Involved in the study | |
|---|---|---|
| ☒ | ☐ | Antibodies |
| ☒ | ☐ | Eukaryotic cell lines |
| ☒ | ☐ | Palaeontology and archaeology |
| ☒ | ☐ | Animals and other organisms |
| ☒ | ☐ | Clinical data |
| ☒ | ☐ | Dual use research of concern |

## Methods

| n/a | Involved in the study | |
|---|---|---|
| ☒ | ☐ | ChIP-seq |
| ☒ | ☐ | Flow cytometry |
| ☒ | ☐ | MRI-based neuroimaging |
